# Application of Microfluidics in Drug Development from Traditional Medicine

**DOI:** 10.3390/bios12100870

**Published:** 2022-10-13

**Authors:** Xue Li, Xiaoming Fan, Zhu Li, Lina Shi, Jinkuan Liu, Hongzhi Luo, Lijun Wang, Xiaoxin Du, Wenzhu Chen, Jiuchuan Guo, Chenzhong Li, Shan Liu

**Affiliations:** 1 Sichuan Hanyuan County People’s Hospital, Hanyuan 625300, China; 2 Department of Laboratory Medicine, Sichuan Academy of Medical Sciences and Sichuan Provincial People’s Hospital, University of Electronic Science and Technology of China, Chengdu 610072, China; 3 College of Medical Technology, Chengdu University of Traditional Chinese Medicine, Chengdu 610075, China; 4 School of Medicine, University of Electronic Science and Technology of China, Chengdu 610054, China; 5 Department of Laboratory Medicine, The Third Affiliated Hospital of Zunyi Medical University (The First People’s Hospital of Zunyi), Zunyi 563002, China; 6 Department of Ophthalmology, The Third People’s Hospital of Chengdu, The Affiliated Hospital of Southwest Jiaotong University, Chengdu 610031, China; 7 Office of Scientific Research & Development, University of Electronic Science and Technology, Chengdu 610054, China; 8 Department of Blood Transfusion, The First People’s Hospital of Longquanyi District, Chengdu 610041, China; 9 The M.O.E. Key Laboratory of Laboratory Medical Diagnostics, The College of Laboratory Medicine, Chongqing Medical University, #1 Yixueyuan Road, Yuzhong District, Chongqing 400016, China; 10 Department of Biochemistry and Molecular Biology, School of Medicine, Tulane University, New Orleans, LA 70112, USA; 11 Sichuan Provincial Key Laboratory for Human Disease Gene Study, Department of Medical Genetics, Sichuan Academy of Medical Sciences & Sichuan Provincial People’s Hospital, University of Electronic Science and Technology of China, Chengdu 610072, China

**Keywords:** microfluidics, drug development, traditional medicine, bioMEMS

## Abstract

While there are many clinical drugs for prophylaxis and treatment, the search for those with low or no risk of side effects for the control of infectious and non-infectious diseases is a dilemma that cannot be solved by today’s traditional drug development strategies. The need for new drug development strategies is becoming increasingly important, and the development of new drugs from traditional medicines is the most promising strategy. Many valuable clinical drugs have been developed based on traditional medicine, including drugs with single active ingredients similar to modern drugs and those developed from improved formulations of traditional drugs. However, the problems of traditional isolation and purification and drug screening methods should be addressed for successful drug development from traditional medicine. Advances in microfluidics have not only contributed significantly to classical drug development but have also solved many of the thorny problems of new strategies for developing new drugs from traditional drugs. In this review, we provide an overview of advanced microfluidics and its applications in drug development (drug compound synthesis, drug screening, drug delivery, and drug carrier fabrication) with a focus on its applications in conventional medicine, including the separation and purification of target components in complex samples and screening of active ingredients of conventional drugs. We hope that our review gives better insight into the potential of traditional medicine and the critical role of microfluidics in the drug development process. In addition, the emergence of new ideas and applications will bring about further advances in the field of drug development.

## 1. Introduction

Although drugs are currently available for the treatment and control of common health problems, including high blood pressure, diabetes, AIDS, diarrhea, and tumors, there is a global concern for the development of drugs with low or no side effects for the control of contagious and non-contagious diseases. These conditions continue to affect diverse human populations around the world with significant mortality. Current drug development strategies do not address this problem; hence, new drug development strategies are needed [1]. At present, the new drug development strategy with the greatest potential to meet demand is to develop new drugs from traditional medicine. Traditional medicine actually refers to a long history of treatment methods such as Traditional Chinese Medicine, Ayurveda, and Iranian Traditional Medicine. The means of overcoming diseases through natural medicines occupies a huge part of it. Globally, there is a long history of controlling diseases through the use of natural medicines, and traditional medicine is not only effective but can be obtained directly from existing natural medicines or by modifying traditional medicine formulations [2,3,4]. About a quarter of all drugs authorized by the U.S. Food and Drug Administration (FDA) and/or the European Medicines Agency (EMA) are substances of plant origin, including highly effective drugs such as paclitaxel, morphine, and artemisinin [1]. Other examples are Dan Dihuang Tang, Qi Tang, and Easy Kidney Capsules, which have been effectively used in clinical practice to prevent diabetic nephropathy [5]. However, the process is not straightforward, and there are at least two obstacles to developing new drugs from traditional medicine. One challenge is the need for methods that can efficiently separate and purify components from complex samples; the second challenge is the urgent need for reliable new drug screening protocols because of the inadequacy of traditional drug screening protocols.

First, the primary obstacle is the separation and purification of target components from complex samples. The development of traditional drugs with single active ingredients is highly desirable [3]. However, since traditional medicines usually contain multiple compounds and are complex, extraction and purification are required. New drugs can also be developed by improving traditional drug formulations. However, this method suffers many setbacks because differences in the geographical origin of herbs, growing conditions, agricultural production methods, and drug production processes may lead to differences in the chemical composition and therapeutic effects of the final product [2]. Harmful additives, such as formaldehyde, may also be incorporated during the preparation process [6]. Therefore, to ensure the safety, efficacy, and consistency of drugs, there is a need to control their quality. The current quality control method mainly involves the detection of specific components in the sample; however, the complex composition of the sample does not favor the detection system because it impacts the test results. Therefore, pretreatment of the sample for separation and purification is essential for more reliable results. In addition, the overdose of some dangerous drug components may cause adverse consequences; hence, real-time monitoring of body concentrations of drugs is required. However, body fluid samples usually contain several contaminants, and the target drugs are often present in the samples at low concentrations [7]. Thus, isolation and purification are important elements to be considered, especially because traditional means of separation and purification do not meet scientific standards.

Liquid–liquid extraction (LLE) is a classical approach to the separation and purification of target compounds in complex liquids (body fluids and traditional drug crude extracts) as a sample pretreatment step. LLE enables the stepwise separation of active ingredients through sequential extraction with different organic solvents to obtain different compounds for subsequent activity screening [8]. However, LLE has various disadvantages, including emulsion formation, low extraction efficiency, time consumption, high use of organic solvents and samples, difficulties in automation, production of large amounts of toxic solvents, and the release of waste into the surrounding [3,5,8]. Therefore, to achieve a breakthrough in the development of new drugs in traditional medicine, the separation and purification process should have fewer operation steps and be more efficient, environmentally friendly, and automated.

The adoption of microfluidics may be an effective way to achieve most of these goals. Laminar flow is a unique phenomenon in the micro-sized structure of microfluidic chips. When two immiscible liquids enter the same channel simultaneously from separate inlets, a stable interface with a high surface area is usually found between the stratification of the two fluids due to the small Reynolds value of the microfluidic channel. Molecules can move rapidly from one liquid to the other by diffusion and affinity. The combination of LLE and microfluidics improves extraction efficiency, reduces processing time, and lowers the cost of reagents [3,7,9,10,11,12]. Although toxic solvents may still be required, toxic solvents and waste emissions are reduced indirectly because of the improved extraction efficiency. In addition, microfluidic chips are easy to automate, integrate, and parallelize [7,10,11]. Therefore, the use of microfluidic chips is advantageous for the separation and purification of complex samples.

Secondly, while developing a drug with only one active ingredient or modifying a traditional formulation to develop a drug, an important step that cannot be bypassed is drug screening. Traditional screening methods include in vitro cellular assays, in vivo animal experiments, and human clinical trials. However, the process involves significant human and financial resources, as well as time [13]. It is apprehensive that both traditional in vitro cellular models and in vivo animal models have many drawbacks. For example, the common in vitro cell model, MTT, is based on cytotoxicity analysis with a microtiter plate based on a cell colorimetric method. It is not only time consuming but also usually involves the consumption of large amounts of expensive and not easily available herbal components. Since the test uses a colorimetric method, antioxidants and colorants may alter its reliability and sensitivity. In addition, the inability to replicate changes in the in vivo microenvironment may result in unpredictable in vivo effects of the drug. In vitro animal models also face considerable challenges. The high cost of experimental animals, the difficulty of handling them, the difficulty of genetic and physiological differences in humans, and the inevitable ethical issues pose obstacles to drug development. Moreover, the therapeutic activity of traditional medicine is often due to the synergistic and simultaneous action of several chemicals, and therefore often requires the testing of a large number of chemical combinations and relies on data at multiple qualitative and quantitative levels [1]. These are some of the difficulties that need to be addressed regarding the screening process of candidate drugs when drug development is carried out from traditional medicine. Thus, the chosen screening methods should be easy to operate and able to replicate the human in vivo environment; they should also be rapid, high throughput, low cost, reliable, and sensitive [13,14,15,16]. The application of microfluidics to drug screening can provide a solution to these challenges. As microfluidic devices are easy to miniaturize, automate, integrate, and parallelize, they reduce the consumption of reagents, shorten reaction time, improve throughput, reduce waste generation, and simplify operational steps. Notably, microfluidics can meet the need for accurate modeling of physiological conditions on the same device and can simulate in vivo microenvironments at the single cell/2D, 3D, and organ/human level, thus addressing the shortcomings of in vivo and ex vivo models in the drug screening process. In summary, the application of microfluidics to the screening of traditional medicines is not only simple and environmentally friendly but can also improve research efficiency, shorten the research cycle, reduce research costs, and improve the success rate of drug development. Thus, it can be employed to solve the potential problems associated with the screening of in vitro models of drugs in vivo [16,17,18,19,20,21,22,23].

In summary, as a new drug development strategy, the development of new drugs from traditional medicine has an unparalleled prospect and has been quite fruitful. Recent interest in microfluidic technology is justified owing to its wide range of applications and advantages. However, there is a dearth of information on this technology. This review briefly describes the development of microfluidics and their applications in three areas of drug development (drug compound synthesis, drug screening, drug delivery, and drug carrier manufacturing). We focus on the application of microfluidics to traditional medicine for drug development in two areas: purification and separation of sample constituents from complex products and screening of active ingredients in traditional medicines. This review is expected to provide new reflections and contributions to the modern utilization of traditional medicine and the promotion and application of microfluidic technologies.

## 2. Microfluidics

According to Perkel, microfluidics is the study of moving and manipulating minuscule quantities of fluids through nanoscale channels [24]. Microfluidic chip technology integrates laboratory samples, dilution, reagent addition, reaction, separation, and detection operations on a microchip; it is also known as a micrototal analysis system (TAS) or “laboratory on a chip” [13].

Microfluidics has evolved rapidly in just a few decades. As early as 1965, Richard Feynman, winner of the Nobel Prize in Physics, foresaw a future in which micro- and nano-scale objects were manipulated and utilized [15]. The invention of capillary electrophoresis and flow injection separation on flat microchips by Manz et al. marked the beginning of the microfluidic chip. Glass or silicon was used to construct the first microfluidic device. Glass materials have the advantages of strong insulation, high thermal conductivity, favorable transparency, inexpensive cost, and permanent electrical permeability migration rate, while silicon materials have the advantages of special thermal conductivity, low transparency, and corrosion resistance.

Glass or silicon microfluidic devices can withstand high temperatures and pressures, making them suitable for extraction, biological processes, and material creation. However, the application of these devices was limited by their complexity, high production cost, toxic byproducts, difficulty in mixing multiple components, and restricted gas permeability. Later on, soft lithography for chip production emerged, and microfluidic technology entered a period of rapid development [25,26]. Soft lithography enables the fabrication of various polymer-based microfluidic devices. Polymers are available in a vast array of forms based on their required characteristics. The most common means of fabricating microfluidic chips from dimethylsiloxane (PDMS) is through soft photolithography. In 2002, Whiteside GM et al. invented a microfluidic chip in which PDMS is used on a mold with a predetermined channel structure and molded by soft lithography, resulting in a microfluidic chip with the intended channel. This method is economical, and straightforward and does not require high temperatures or pressures. PDMS equipment specifically addresses the issues associated with glass and silicon material coupling, hazardous substances, gas permeability, and biocompatibility [27]. In addition, microfluidic chips have two characteristics: first, the fluid can accurately and rapidly perform heat transfer and diffusion reactions due to microfluidic effects, such as laminar flow, surface tension, and capillary effect; second, microfabrication technology can handle small, high-density microstructures, allowing flexible combinations and scale integration of various operating units. This gives microfluidic chips the advantages of miniaturization, automation, integration, portability, low energy consumption, rapid detection of sample pre-treatment, and subsequent analysis [12]. These advantages contribute to the irreplaceable benefits of microfluidic chips for many research applications, such as chemical synthesis, proteomics, single-cell analysis, tissue engineering, high throughput screening, environmental analysis, and medical diagnostics [28]. In July 2006, Nature published six articles introducing microfluidics and highlighting its importance in biomedical and pharmaceutical research [13]. Numerous drug discovery processes can benefit from the use of microfluidics, including drug compound synthesis, screening, delivery, and carrier manufacturing. As well, with the advent of smart microfluidics in recent years, the paradigm of manually manipulating microfluidic devices and performing experimental data analysis has gradually changed. This section consists of a concise overview of the application of microfluidics in these fields.

### 2.1. Drug Compound Synthesis

Drug compounds are synthesized either to obtain a medicinal and biologically significant product or to modify an existing compound to make it more clinically valuable [26,29,30,31]. Drug discovery involves designing the molecular structure of the drug, lead compound synthesis, and lead optimization for pharmacological or clinical application. Existing compounds can also be used as lead compounds, and the optimization of their pharmacology or clinical application cannot be missed [32,33]. Thus, the entire process of drug synthesis involves synthetic chemistry. Traditionally, chemical synthesis is uncontrollable, inefficient, error prone, and requires large amounts of time and reagents. The advent of microfluidic technology offers benefits, such as less reagent use, faster reaction speeds, lower embodied expenditures of energy, smaller equipment, greater selectivity of reaction concerning products, and more secure reactions [26]. This section describes microreactors for drug synthesis, such as the microchannels reactor and the droplet microreactor.

#### 2.1.1. Microchannel Reactor

**Figure 1:**
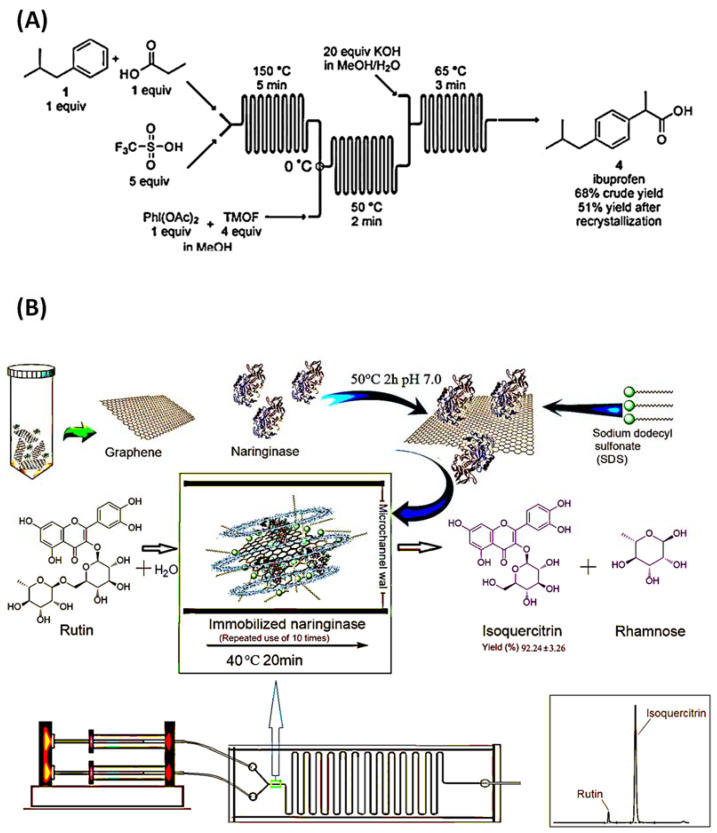
Schematic diagram of drug synthesis by Microchannel reactor: (**A**) Schematic diagram of the biosynthesis of ibuprofen in a microchannel reactor from [26], Copyright 2021, American Chemical Society. (**B**) Schematic diagram of the biosynthesis of isoquercitrin in a microchannel reactor from [35], Copyright 2017, Nature Publishing Group.

Microchannel reactors have been used for decades. A microfluidic chip for the synthesis of ibuprofen was fabricated in 2009. On this microfluidic chip, there are three reactors, each responsible for a separate chemical reaction step. In this microfluidic chip, a three-step chemical reaction is performed by a three-step continuous flow, and finally, ibuprofen is synthesized. The results show that the yield of crude ibuprofen can reach 9.8 mg/min, with 68% crude yield and 51% recrystallization yield, as shown in Figure 1A [26,34]. In a new development, Gong et al. proposed a method for achieving productive and reproducible biocatalytic reactions in microchannels using graphene sheet-immobilized naringenin enzymes to optimize the production of isoquercitrin in microchannel reactors. This research can also be applied to other drug synthesis reactions that require enzymes or catalysts. However, it is evident that multi-step conversion is necessary for most complex molecules, and after specific designs, the microchannel reactor is also suitable for multi-step conversion, as shown in Figure 1B [35]. Apart from a microchannel reactor, which is an effective use of microfluidics in drug synthesis, a droplet microreactor is also a decent method for applying microfluidics in the drug synthesis process.

#### 2.1.2. Droplet Microreactor

**Figure 2:**
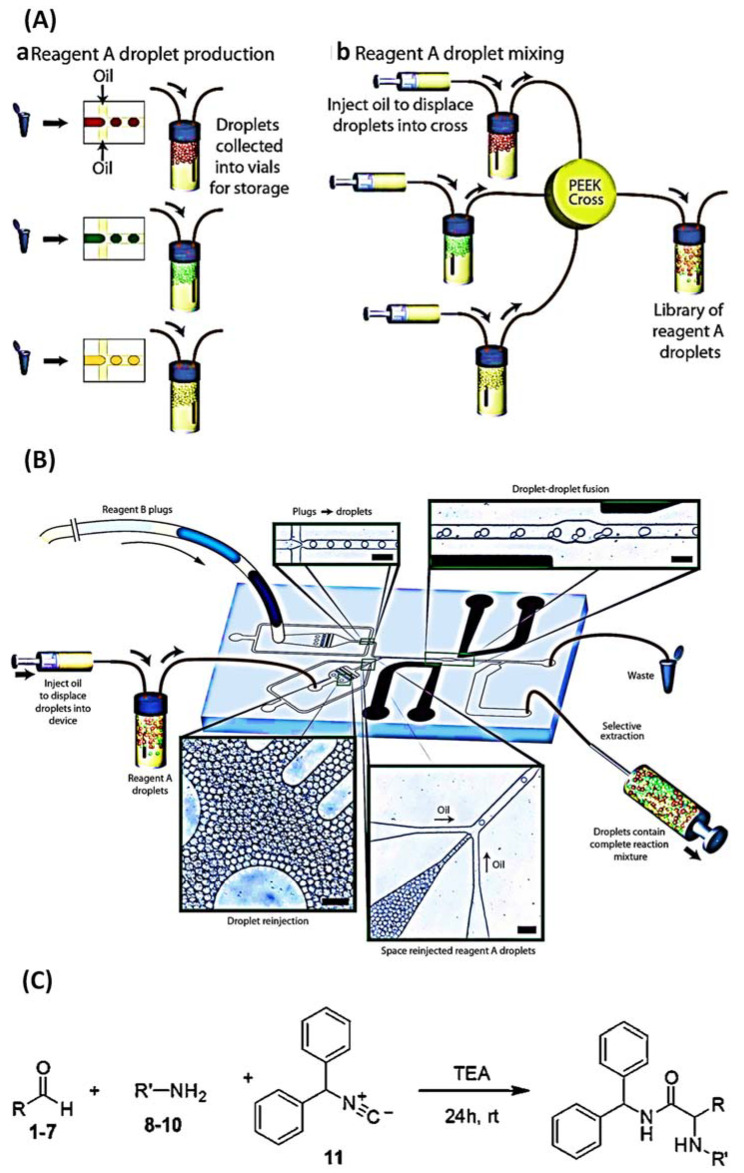
Schematic representation of the synthesis of active molecules with the potential to inhibit thrombin by a Droplet microreactor: (**A**) Schematic diagram of the reagent A droplets. (**B**) Schematic diagram of chemical synthesis by injection of microdroplets. (**C**) Schematic representation of the synthesis of molecules with potential prothrombin-inhibiting activity by amines, aldehydes, and isocyanides. The image from [38], Copyright 2012, Royal Society of Chemistry.

In fact, there are many ways to generate droplets, such as valves, CNC, electric fields, magnetic fields, acoustic waves, syringe pumps, and gravity. It is entirely possible to produce suitable droplets in different ways according to the required requirements [36]. Microfluidic droplet microreactors offer numerous benefits, such as replacing tedious biochemical laboratory operations, reducing the reaction generating area, performing high flux reactions, efficient conduction, stabilizing the product quality, and synchronizing the reaction time. The in situ reaction is a popular method of droplet chemical synthesis: all reactants are synthesized into droplets before the reaction [26]. Sarah L. Poe et al. invented a method for droplet collision diffusion using microfluidic chips. The principle of droplet collision is that none of the disperse-phase reagents can be mixed with the carrier phase, so the reagents are mixed in the tube by droplet collision. These droplets can then be used as separate reactors for chemical reactions, and the reaction products are confined in these droplets. Therefore, after the reaction occurs, the product will be far away from the tube wall, and the purpose of avoiding the deposition of reaction products in the microfluidic device will be achieved. It was found experimentally that small droplets centered in the oil tube were produced in this device when the carrier phase was mineral oil and the flow rate was 3 mL/min. They used the indigo synthesis reaction to verify the effectiveness of this anti-sink design due to the precipitation and color change that occurs with the indigo synthesis reaction. The results demonstrate that the use of mineral oil as a carrier at a flow rate of 3 mL/min can achieve anti-deposition [37]. Another form of trigger synthesis exists in which the reaction can begin at a predetermined moment. As an example, a closed droplet microfluidic chip capable of immobilizing compounds was studied using PDMS as the material. The Ugi-type reactions of amines (A1–A3) and aldehydes (B1–B7) with isocyanide were carried out on this chip. Small molecules that can inhibit thrombin were synthesized, as shown in Figure 2. A monodisperse droplet in a droplet microfluid acts as a standalone microreactor that can perform a diverse range of reactions in tailored environments. Regardless of the reaction mode used, it allows for universal reagent selection and minimal specimen depletion [38].

### 2.2. Drug Screening

Drug compound synthesis is only the first step in the development of classical drugs. Drug screening is a critical step in determining the value of a candidate drug. The drug screening process screens potential drugs for efficacy, cytotoxicity, and adverse effects. Only after these series of trials can the drug be confidently made available for use in the clinic. However, it takes an average of 10 to 15 years to complete this process of developing a chemical into a new drug and getting it into the market, and costs between USD 1.5 and 1.8 billion. Therefore, efficient and low-cost drug screening methods are urgently needed [28]. Microfluidic chip analysis technology can condense the steps involved in sample pretreatment, reaction, derivation, separation, and detection onto a single chip and reduce the analysis time in the form of fluid and array multichannel in comparison to the conventional multi-plate detection system. It provides a conducive environment for cell physiological and biochemical research with minimal sample utilization. Particularly with the advent of microfluidic technology, the limitations of 3D chips, organ chips, and more complex human chips in supplementing single-cell chips and 2D chips have become evident [13,28,39,40,41]. This section will shortly describe the application of these microfluidic chips in drug screening, including single-cell and 2D, 3D, and organ and more complicated human chips.

#### 2.2.1. Single-Cell and 2D Chips

Droplet microfluidics is one of the most prevalent techniques for encapsulating single cells; single-cell droplets are suitable for high-throughput screening and sorting. As they can circumvent the average population and make room for intercellular heterogeneity, single-cell microarrays have become the preferred tool for the screening of potential drugs [15,25,42,43,44,45].

**Figure 3:**
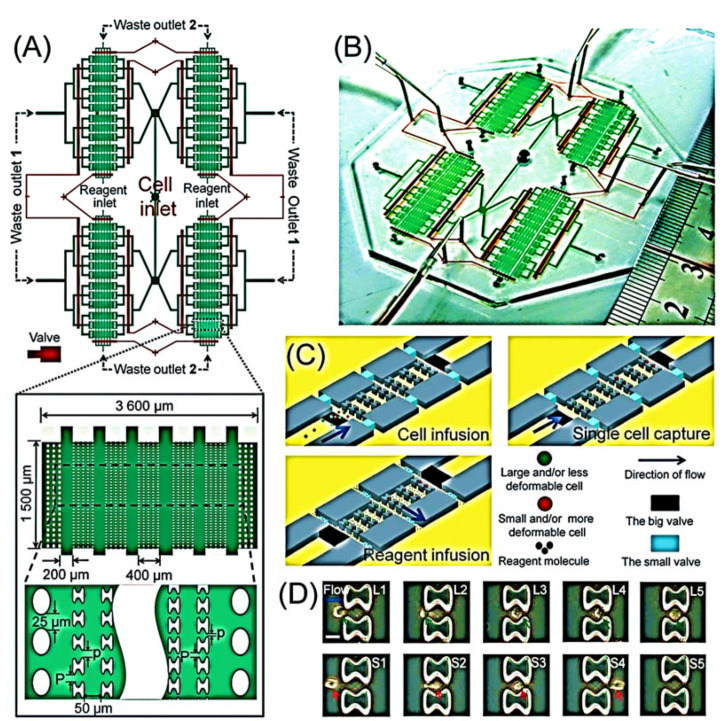
Schematic diagram of the Microfluidic chip for obtaining single cells: (**A**) Schematic diagram of the design of the Microfluidic chip. (**B**) Actual image of the Microfluidic chip, measuring 6.0 cm × 5.0 cm. (**C**) Schematic diagram of the operation of single cell sorting and reagent infusion. (**D**) Schematic diagram of the typical dynamic processes of single cells captured by (L1–L5) or passed through (S1–S5) filter cells. The image from [39], Copyright 2016, Royal Society of Chemistry.

**Figure 4:**
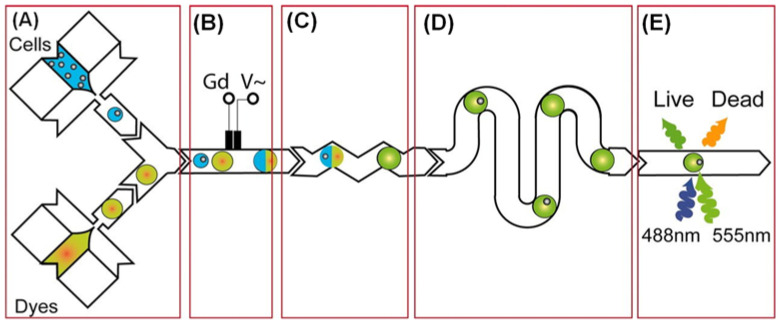
Schematic diagram of drug screening by Droplet Microfluidic Chip: (**A**) Inject droplets containing cells and fluorescent dye. (**B**) Droplet merging is controlled by electricity. (**C**) Mixing dyes with cells. (**D**) Optimize cell staining by extending channels. (**E**) Collect fluorescent signals. The image from [46], Copyright 2009, PNAS.

**Figure 5:**
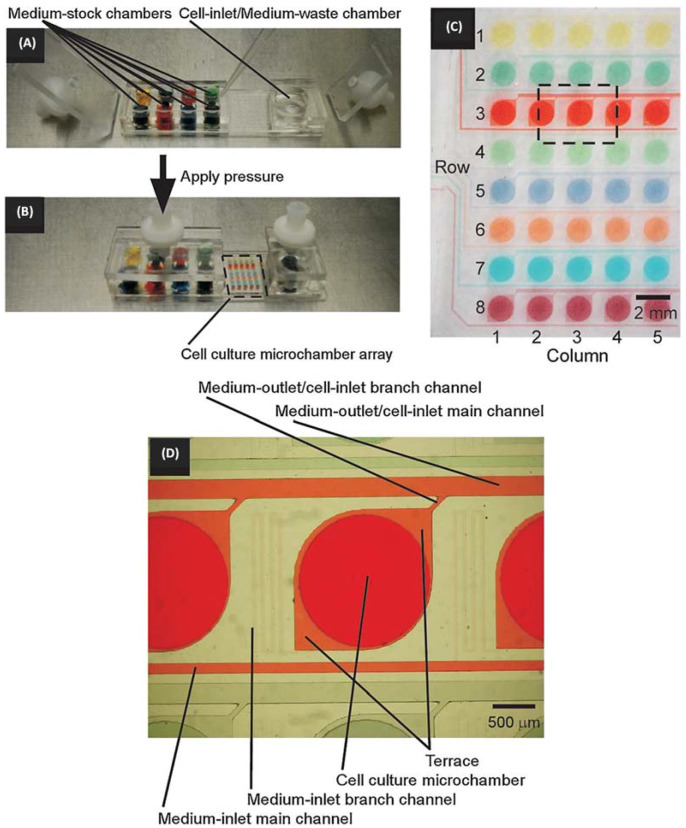
Schematic diagram of a Microfluidic chip driven by pressure: (**A**) Eight different dyes are injected into the Microfluidic chip. (**B**) Pressure is applied by venting from the outside through a sterile ventilation filter, with the dashed line indicating the enlarged area in (**C**). (**C**) Microscopic photograph of the Microfluidic chip, with the dashed square indicating the expanded region in (**D**). (**D**) Three colors indicate three different microstructures. The image from [42], Copyright 2008, Wiley.

Long Pang et al. created an integrated microfluidic device that can capture single cells based on their biomechanical characteristics (size and deformability) and analyze their drug resistance, as shown in Figure 3 [39]. Brouzes et al. developed a droplet viability assay that allows for the quantitative scoring of cell viability and proliferation within intact droplets, enabling the screening of cytotoxic effects of the entire drug library on the cells, as shown in Figure 4 [15,46]. Shinji Sugiura et al. described a pressure-driven perfusion culture chip for parallel drug cytotoxicity assays. The device comprised an 8 × 5 array of microchambers for cell culture with independent perfusion microchannels, permitting uniform cell loading and perfusion culture without cross-contamination between adjacent microchambers. They evaluate the cytotoxic effects of seven therapeutic drugs in parallel using this microfluidic chip. The capability of the chip to easily eliminate air bubbles, inject uniformly, and quantify cell proliferation makes it an efficient method for screening potential drug candidates (Figure 5) [42].

**Figure 6:**
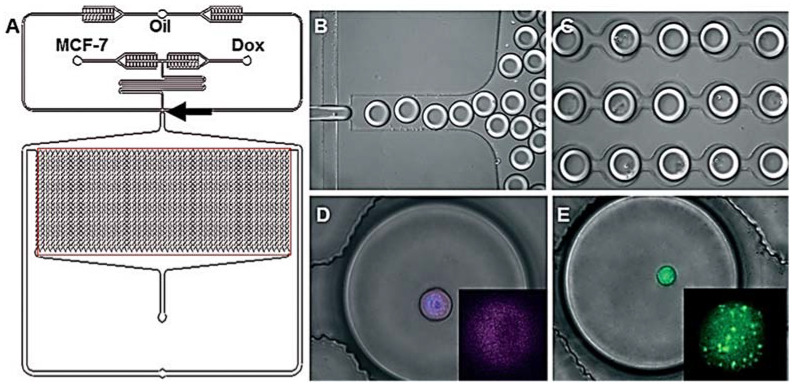
Schematic diagram of single-cell encapsulation in droplets by Microfluidic chip: (**A**) Microfluidic chip, arrows are droplet generation junctions, and boxes indicate droplet docking arrays. (**A**) Microfluidic chip, arrows are droplet generation junctions, and boxes indicate droplet docking arrays. (**B**) Droplet generation. (**C**) Microdroplet docking. (**D**) Detection of Cy-5 conjugated ABCB-1 mRNA in live MCF-7S cells in droplet (enlarged in inset), Hoechst labeled cells nuclei. (**E**) Dox-resistant MCF-7R cell encapsulation in droplet. Inset: Calcein AM localization in vesicles. The image from [47], Copyright 2015, Royal Society of Chemistry.

Due to the potential advantages of reduced drug toxicity and enhanced therapeutic efficacy of drug combination therapy, a growing number of studies are being conducted on drug combination use. Guan-Sheng Du et al. described cell-based drug combination screening using a microfluidic droplet system based on a sequentially operated droplet array (SODA) technology. The system incorporates multiple steps, including long-term cell culture, media changes, schedule-dependent drug dosing, stimulation, and cell viability testing [43]. Sabhachandani et al. encapsulated individual MCF-7 breast cancer cells and adriamycin in an integrated microliter droplet array of microfluidic droplets. The cell suspension was incubated through one inlet and the adriamycin medium through another inlet to generate monodisperse droplets at an optimized flow rate, as shown in Figure 6. The device is designed to contain a 2.05 cm long serpentine segment before the droplet generation connection to promote the lateral alignment of cells. These droplets are subsequently oriented into a parallel docking array that consists of optimally spaced contiguous capture sites to prevent contact-mediated merging over longer periods. The capture sites are numbered to facilitate the dynamic monitoring of identical droplets, thus allowing the analysis of identical individual cells in a population rather than different individual cells [47].

#### 2.2.2. 3D Chips

Cell array technology, based on two-dimensional cell cultures, cannot recreate the natural in vivo environment required for normal cell evolution and migration. In contrast, 3D tissue models provide cell–cell and cell–extracellular matrix interactions, as well as the diversity of space and physical chemistry, all of which have a substantial effect on disease. In addition, they provide a platform for scalable, high-throughput drug screening systems and the ability to identify the structure–function link and recreate cell research and disease progression. The size of the three-dimensional cell spheroid can be easily controlled by modifying the geometry of the cell culture chamber of the device. The 3D cell model correctly manages fluid through micron-sized channels. The coupling of three-dimensional cell culture with a microfluidic network on a microchip may help the development of tissue-like structures in vivo, given the information presented above [28].

**Figure 7:**
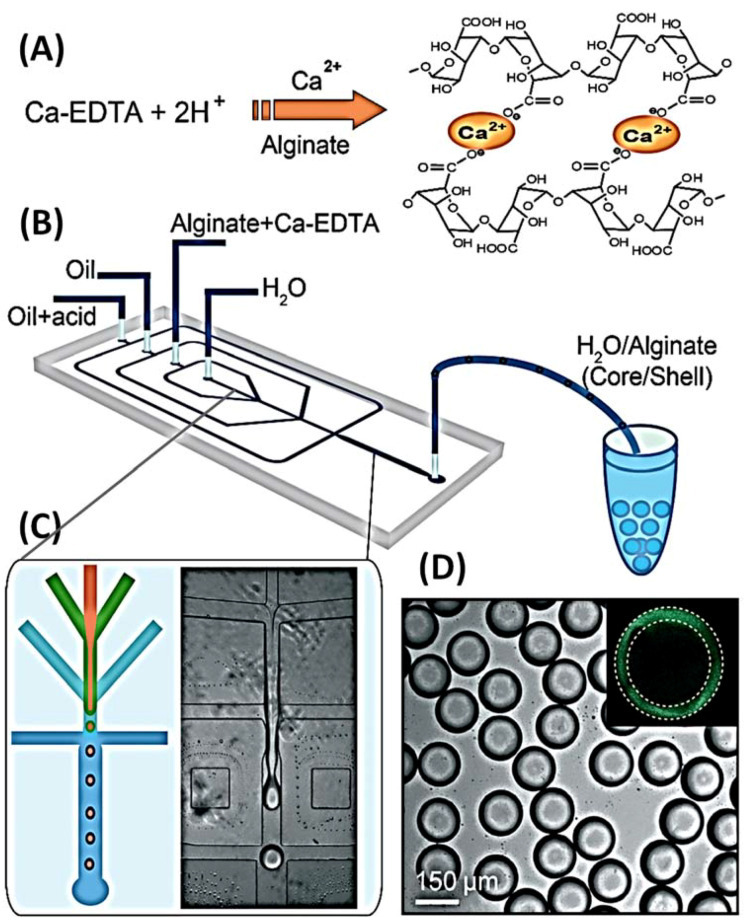
Schematic diagram of the creation of a three-dimensional scaffold in a droplet (composed of a water nucleus and a hydrogel shell): (**A**) Principle of obtaining alginate networks. (**B**) PDMS Microfluidic chip. (**C**) Alginate cross-linking in the Microfluidic chip. (**D**) Fluorescein-labeled alginate as shown in the inset where the shell of alginate hydrogel was found under confocal microscopy. The image from [48], Copyright 2016, Royal Society of Chemistry.

The most prevalent technique for 3D cell culture entails encapsulating cells in 3D hydrogel materials, such as collagen, agarose, and various synthetic hydrogels. Hydrogels imitate the interaction of cells with the matrix. The high permeability of hydrogels enables the diffusion and exchange of oxygen, nutrients, and metabolites, thereby sustaining the growth and function of cells [41]. Qiushui Chen et al. employed dual emulsion as a template for the creation of core-shell drops in a stream microfluidic chip, as illustrated in Figure 7. The inner phase is a cell culture medium, and the middle phase is an aqueous alginate solution, a well-studied biocompatible polymer that showed good cell function and viability in the network. Due to the low Reynolds number, the inner phase co-flows with the middle phase. The oil forms monodisperse droplets at the intersection consisting of an aqueous core and a hydrogel shell (Figure 7B,C). Since UV irradiation is usually harmful to the cells, it is not a good method for crosslinking. The innovative method of introducing an additional oil stream containing 0.15% acetic acid downstream and triggering the release of Ca^2+^ from the Ca-EDTA complex in alginate was used. Subsequently, divalent Ca^2+^ binds to two different carboxyl groups of the alginate chain, forming a crosslinked three-dimensional network, as shown in Figure 7A. In situ cross-linking locks the alginate in the shell, as shown in Figure 7D [48].

Zhuhao Wu et al. recently developed a novel microfluidic device for fabricating scaffold-free 3D microspheres for breast tumor models using an innovative acoustic fluidics technique. The 3D microspheres fabricated by this device are not only homogeneous and fast to fabricate but also suitable for a wide range of cell types. In fact, although ordinary acoustic currents can aggregate cells into spheroids, they produce few spheroids and vary in size. The controlled, stable, low thermal effect and biocompatible properties of a surface acoustic wave make it a powerful player in the fabrication of 3D microspheres. In this experiment, only a cell suspension is loaded on the PDMS device and the acoustic radiation force is applied to the suspended cells to create microspheres with good results. More definitely, by changing the concentration and ratio of cells in the suspension, the size and cellular composition of the spheroids can be adjusted, and the growth process of the spheroids can be clearly monitored. All in all, this attempt was very successful and showed the great potential of acoustic fluids for 3D cultures [49].

Brooke et al. further developed an automatable 3D culture and analysis platform on microfluidics. The platform not only enables individual, combinatorial, and sequential drug screening, but also real-time analysis. Firstly, automation not only reduces the workload, but also allows accurate control of the reagent addition process through programming, including the moment of drug addition, the amount of drug addition, and the order of drug addition. The implementation of automation greatly avoids the errors associated with manual intervention and enables the reliable determination of drug effects. Next, the advantage of 3D culture and organoid systems over traditional two-dimensional platforms is that they provide more accurate biological information. This is because 3D cultures and organoid systems can better simulate the physiological state of cells and tissues in vivo. Lastly, the real-time analysis enables the most intuitive and rapid discovery of drug effects and the determination of the most appropriate dosing regimen. It is worth acknowledging that the experiment draws on an automated 3D microfluidic platform to provide direction for personalized dosing or screening of effective drugs. The experiments showed significantly different results for different types of drugs, different types of drug combinations, and different doses of drugs, which provides the possibility to explore the most appropriate dosing regimen. In addition, this platform is sure to shine when applied in screening effective drugs from potential drugs. The convenience, accuracy, and physiological advantages of the automated 3D microenvironment, as well as the timeliness and intuitiveness of real-time monitoring, can help us pinpoint effective drugs and even delve further into their pharmacodynamics [50].

#### 2.2.3. Organ Chips and More Complicated Human Chips

An organ chip is a system that simulates the microstructure and physiological functions of specific human organs using Bio-MEMS or microfluidics and represents the essential functional units of human organs. The objective of the drug-on-a-chip screening system is to create an organ-specific disease model on an organ chip and then determine the pharmacological activity or biological toxicity of drugs [28,41]. Currently available organ-based devices include liver, lung, and heart chips. In addition, there have been numerous exhaustive reviews, as shown in Table 1.

**Figure 8:**
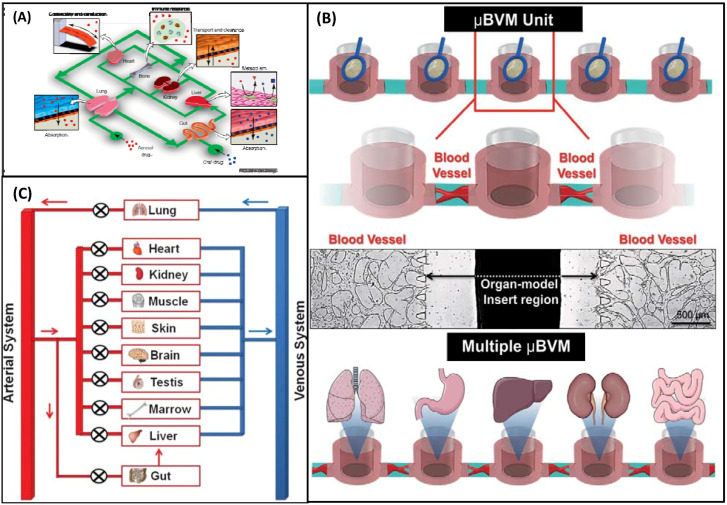
Schematic of a human on a microfluidic chip: (**A**) An integrated system composed of micro-organs. The image from [51], Copyright 2011, Elsevier. (**B**) Integrated system of multiple culture dishes connected by perfusable vessels. The image from [52], Copyright 2015, SAGE. (**C**) Integrated system of different organ-on-a-chip couplings. The image from [53], Royal Society of Chemistry.

Moreover, a human body chip to simulate organ and tissue functions by integrating the functions of multiple organs on microfluidic devices is proposed. Human chips simulate the pharmacokinetics and pharmacodynamics of drugs entirely in vitro, with the potential to replace animal models due to concerns regarding cost and ethics. Ingber (Figure 8A) [51], Ryu (Figure 8B) [52], and Huh (Figure 8C) [53] led their respective teams in their efforts to create a human chip. These human chips may crack the traditional drug screening model puzzle and serve to unlock the drug delivery dilemma.

### 2.3. Microfluidics for Drug Delivery and Drug Carrier Fabrication

In drug development, the impact of the method of administration must be considered. Common methods of drug delivery include digestive system absorption, dermal system injection, and respiratory system inhalation. These methods are likely to lead to adverse reactions in patients if the amount, effect and duration of action of the input drug are not controlled. In addition, there are medications whose actions are weak and easily altered by the receptor environment, making them unsuitable for conventional delivery methods [26]. Therefore, it is crucial to develop drug delivery systems that can modulate the entry of drugs into the body for action. The development of microfluidic drug delivery systems or the fabrication of microcarriers for drug delivery and controlled release by microfluidics are potentially valuable. This section describes several microfluidic micropumps and microfluidic-based carriers.

#### 2.3.1. Microfluidic Micropumps for Drug Delivery

Micro-nano-controlled fluid delivery capability and the ability to integrate with multiple additional subsystems make the micropump useful in a variety of drug delivery scenarios. In general, micropumps can be separated into two categories based on their driving mechanisms: driven miniature valves (both mechanical and non-mechanical miniature valves) and non-driven miniature valves. Firstly, Mechanical micropumps produce pulsing flow by mechanically moving components, such as reciprocating pistons, vibrating diaphragms, or spinning gears and blades. In the pumping cycle, these movable structures actuate fluid motion [54]. Electric, thermal, and magnetic micropumps are examples of common mechanical micropump driving concepts [26]. Secondly, to drive fluid, non-mechanical micropumps convert non-mechanical energy (such as current power, magnetic current power, electroosmosis, electrochemistry, and biochemistry) to kinetic energy [55]. Therefore, the performance of a non-mechanical micropump is highly dependent on system characteristics, such as the generation of response time, the velocity of a stream, and the actuation capacity. Finally, the unpowered micropump is spontaneously driven by osmotic or environmental stimulation and is independent of the external power supply. Nevertheless, their drug delivery effectiveness is frequently predetermined by their manufacturing materials, processes, configurations, and environmental conditions. Once these micropumps begin operating, it is difficult to modify or suspend them [56,57,58].

#### 2.3.2. Microfluidic Fabrication of Drug Carriers

Protecting the activity of pharmaceuticals and controlling their loading and release is possible using drug carriers. In addition to ultrasonic waves, stirring, spraying, phase separation, and microfluidic technologies, there are several methods for producing drug carriers. Of these, microfluidics is the most optimum method of drug microcarrier production, with effective delivery and favorable release benefits. Microfluid-derived drug carriers are distinguished by their high loading efficiency, continuous adjustable release spectrum, prevention of drug failure, decrease in adverse effects, and enhancement of drug delivery efficiency. Microfluidics has been used to develop a variety of medication carriers in recent times. Four types of drug carriers are introduced based on their shape and physical and chemical properties: emulsion, microparticles, microcapsules, and microfibers [26].

Emulsion: In most cases, a combination of immiscible liquids. Microfluidic technology has made it possible to manufacture emulsion droplets that fulfill expectations.

Microparticles: Include natural and man-made organic and inorganic materials. Typically, microparticle preparation involves the creation of droplet templates followed by solidification. According to the qualities of the material, various solidification techniques, such as optical aggregation, hot gelling, catalyst vaporization, and phase transfer, can be used.

Microcapsules: The inner core space of a microcapsule is a solid shell that can encase drugs. The microfluidization technique was first employed to establish a twin emulsion droplet template. Since then, a variety of homogeneous microcapsules has been prepared using diversified curing techniques.

Microfibers: Made of all types of substances and can have any form [26].

### 2.4. Intelligent Microfluidics

As artificial intelligence has become more and more sophisticated in recent years, more and more innovative designs have emerged, such as intelligent microfluidics. As mentioned earlier, microfluidics has a wide and important application in the field of drug development, and the emergence of intelligent microfluidics has breathed new life into the drug development business. For example, in drug development, intelligent microfluidics can firstly predict and optimize the chemical synthesis of complex molecules; secondly, it can automate experiments through modular robots to reduce workload and risk of errors; finally, it can achieve high throughput in drug screening experiments and assess the efficacy and safety of drugs through machine algorithms. This section briefly introduces three applications of smart microfluidics in the field of drug development.

First of all, for drug synthesis, to accomplish the synthesis of a target organic molecule, it is necessary to be clear about the four elements, i.e., reactants, reaction conditions, reaction process, and reaction products. In the past, empirical calculations and extensive experience were often required to achieve success, and their scalability and automation were not sufficient. Therefore, the development of tools that can predict synthetic routes has become extremely critical. In recent years, computer-aided chemical synthesis has been in full swing, and the underlying principle is to use artificial neural networks to learn from databases and then to predict chemical reactions. For example, a mechanistic reaction prediction, generation of molecular fingerprints, fingerprint-based reaction prediction, multiple fingerprint feature neural-symbolic approach, and edit-based model are all tools that enable the prediction of the synthesis of organic compounds. However, existing scientific databases are still limited, governing more possibilities of machine models.

Second, the implementation of automated experiments has enormous benefits, not only in terms of saving a lot of physical effort but also in terms of avoiding the experimental impact of manual interference. To achieve idealized automated experiments, machines need to be able to train and optimize themselves with experimental data. However, it must be acknowledged that today’s robotic platforms still rely heavily on manual input, training, and validation, and still have a long way to explore.

Finally, drug screening is a highly tedious and complex process. In particular, each high-throughput screening brings a large amount of data in which we need to find relevant or valid information. Especially with the advent of 3D culture, how to simulate the in vivo microenvironment and more physiological information back requires a lot of statistics and calculations. So, machine algorithms will play a crucial role in this. However, the application of intelligent machines is just in its infancy and cannot yet perform operations such as collecting monitoring data in real time and automatically optimizing response conditions [59].

In the above, we briefly exemplify some experiments of microfluidics in the classical drug development process. In classical drug development strategies, Microchannels Reactor and Droplet Microreactor methods can be applied for drug synthesis; drug screening can be performed on single-cell microfluidic chips, 2D microfluidic chips, 3D microfluidic chips, organ chips, and human chips; microfluidics can be used as an effective tool for drug delivery or drug carrier manufacturing; and finally, intelligent microfluidics is just beginning in the field of drug development, and the development prospects are not to be underestimated despite the many difficulties. This fully demonstrates the breadth, criticality, and developmentality of microfluidics in drug development strategies. However, microfluidics has also been instrumental in the development of drug strategies from conventional medicine.

## 3. Application of Microfluidic Chip Technology in Traditional Medicine

As mentioned earlier, microfluidic chip technology has spread throughout every step of the drug development process and plays an integral role, but classical drug development strategies are inadequate. Although numerous drugs have been produced for epidemic diseases, the mortality rate is still high globally [1]. Over time, traditional medicine has been a powerful therapy for many illnesses. Therefore, the development of new drugs from traditional medicine is a promising strategy [2,3,4]. In recent years, new drug development from traditional medicine, either by obtaining the active ingredient directly from existing natural drugs or through direct modification of a traditional drug formulation, has received increasing attention as a powerful way to address this problem. However, there are quite a few hurdles to overcome when trying to develop new drugs from traditional medicine. Fortunately, microfluidic chip technology also plays an irreplaceable role in the strategy of new drug development from traditional medicine and can be applied to all aspects of the drug development process. However, there are currently insufficient reviews on the role of microfluidic chip technology in the development of new drugs from conventional medicine. In this section, we describe the application of microfluidics in two major steps of drug development from traditional medicine: the separation and purification of target components from complex samples and the screening of active components.

### 3.1. Separation and Purification of Target Components from Complex Samples

In response to the strategy for developing a new drug from traditional ones, whether it is to develop a drug containing a single active ingredient, to directly modify a traditional drug formulation to produce a drug, or to monitor drug concentrations in body fluids to track the body, the requirement for the isolation and purification of a single ingredient from a complex sample is inevitable. However, the extraction and purification methods widely used today are shackled. For example, LLE, as a classical sample pretreatment method, can be used for sequential extraction with different organic solvents to gradually separate the active ingredients or obtain different compounds for subsequent activity screening. However, LLE suffers from various drawbacks that limit its application, including emulsion formation, low extraction efficiency, time consumption, high organic solvent and sample use, difficulties in automation, poor exposure to toxic solvents, and discharge of waste into the environment. [8]. Therefore, we need new advances in extraction and purification methods to facilitate the development of new drugs for traditional medicines. The advent and rapid development of microfluidic chip technology in recent decades have greatly improved our current situation. Due to the unique laminar flow properties of microfluidics, the combination with LLE can be used to achieve improved extraction efficiency, reduced sample pretreatment time, lower reagent and sample consumption, and reduced contaminant emissions. Coupled with the ease of automation, integration, and parallelization of microfluidic chips, there are additional benefits of reducing human intervention, increasing throughput, and facilitating interoperability with other equipment [3,7,9,10,11,12]. The laminar flow phenomenon of microfluidics implies that when two liquids are introduced into the main microchannel of a microfluidic chip from separate inlets, the molecules can move rapidly from one liquid to another by spreading due to the low Reynolds number. Therefore, when LLE is combined with microfluidic chip technology, it is more efficient than macro-LLE [3,10,21]. This, coupled with the ease of automation, integration, and parallelism of microfluidic chips, can reduce manual handling, increase throughput, and facilitate the coupling of other devices for additional studies. With the outstanding contribution of microfluidic technology, we have made a qualitative leap in the field of developing new drugs from traditional medicine [10,59,60]. In the next subsections, we will present the exploration of improved facilitation of separation and purification methods by microfluidics in the process of developing new drugs from traditional medicine, including the development of drugs containing a single active ingredient from traditional medicine, quality control of drugs from improved formulations of traditional medicine, and real-time monitoring of drug concentrations in body fluids.

#### 3.1.1. Development of Drugs Containing a Single Active Ingredient from Traditional Medicine

If an attempt is made to develop a drug containing only one active ingredient from traditional medicine, as is the case with most modern drugs, then due to the complexity of traditional medicine, the action of extracting and purifying a single ingredient from a complex sample needs to be completed [3]. However, as mentioned before, traditional separation and purification methods do not support our research, and the application of microfluidic technology has a practical and effective promotion effect. In the following parts, we will present some experimental designs on how microfluidics can be applied in separation and purification methods for the development of drugs containing only a single active ingredient.

**Figure 9:**
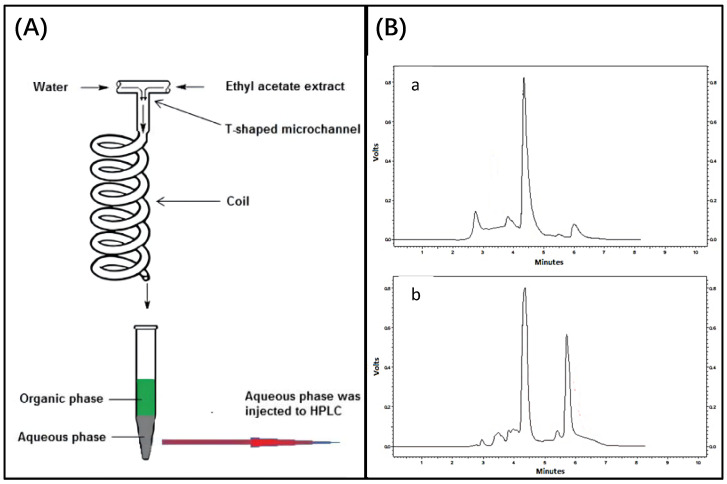
Schematic diagram of the extraction of olive bitter glycosides by Microfluidics: (**A**) Microfluidic device. (**B**) Chromatogram of extracted olive bitter glucoside into aqueous phase: microchannel device (Ba) and ethyl acetate extract (Bb). The image from [5], Copyright 2018, Herbal Medicines Journal.

In 2018, Rouhollah Heydarid et al. attempted to extract olive bitter glycosides from organic extracts of olive leaves into the aqueous phase using microfluidic chip technology (Figure 9). Olive bitter glycosides occupy an important position in medicine and can be used as antibiotics, antimalarial agents, antioxidants, and in the prevention of Alzheimer’s disease. However, the current process of extracting olive bitter glycosides from resins is complex, time consuming, and expensive, making this study of great importance. In this experiment, a syringe pump served as the driving force; the ethyl acetate extract and the aqueous phase entered two separate inlets; a t-shaped microchannel served as the mixing chamber; a coil was provided at the outlet to increase the contact time between the two phases; at the outlet, the two phases were separated because of the difference in density (bottom: aqueous phase and top: ethyl acetate); and the concentration of the two phases of olivetin was determined using a high-performance liquid chromatography (HPLC) system. The principle of the microfluidic device for the extraction of olive bitter glycosides into the aqueous phase using a microfluidic device and ethyl acetate extract is shown in Figure 9A, and a typical chromatogram is shown in Figure 9B [5]. However, two-phase laminar flow microfluidics has an insufficient interface area when compared to three-phase laminar flow microfluidics, which can provide twice the interface area. Under the same conditions, the separation capacity of two-phase microfluidic chips is inferior to that of three-phase layer microfluidic chips [9]. This finding was demonstrated through experiments including the extraction of alkaloids (strychnine and brucine) from plants and two groups of non-polar and polar components with differing clinical effects from *Salvia miltiorrhiza* [8,9]. Taking advantage of the potential benefits of “simultaneous extraction and reverse extraction”, the three-phase chip was developed for the efficient and rapid purification of analytes of interest from drugs and plant extracts at low flow rates.

**Figure 10:**
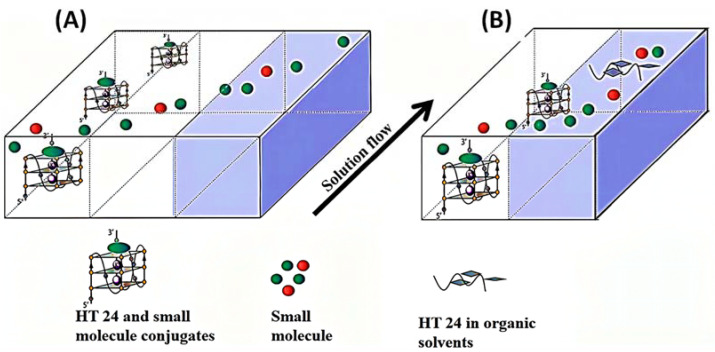
Schematic diagram of the microfluidic chip separating free compounds: three-phase microfluidic chip (**A**) and two-phase microfluidic chip (**B**). The image from [11], Copyright 2020, Elsevier.

In a recent study, Qidan Cai et al. used the g-quadruplex approach to extract strong alkaloids from the extract of Macleaia coral seed using a three-phase laminar flow chip. The mechanism of separation of a free drug and macromolecule by a three-phase chip and a dual-phase chip is described in Figure 10. This experiment allowed a free drug, a G-quadruplex reagent (HT24), and a drug bound to HT24 to enter the TPL chip. Since the free drug has a higher diffusion coefficient, it is more likely to enter the organic phase layer. In contrast, the drug bound to HT24 is left within the sample solution. Finally, the two phases are collected and detected by HPLC. Four antitumor alkaloids, pyrethroids, hesperidin, fisetin, and gallic acid, were eventually obtained by this microfluidic system [11]. Qin Weiwei et al. created two laminar flow extraction devices (three-phase flow microfluidic and continuous r flow microfluidic) on a microfluidic chip. In this experiment, the extraction efficiency of different microfluidic devices was explored by a scheme to isolate and purify ginsenosides from ginseng extract samples, and compared with the current two-phase chip. It was demonstrated that two types of laminar flow extraction can accomplish successive solvent extractions. The extraction efficiency of the successive flow chip was greater than that of the three-phase chip. In addition to the gentle extraction process based on the molecular fusion mechanism, both three-phase chips and continuous chips have the benefit of reducing complex processing and saving time [3].

**Figure 11:**
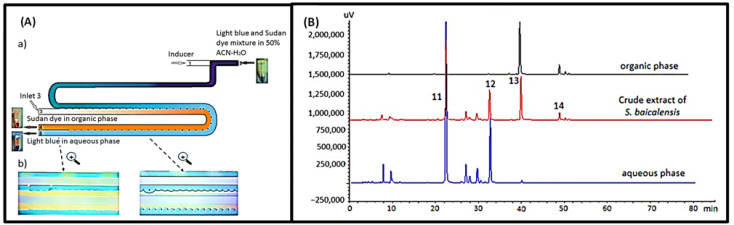
Schematic diagram of the isolation of Scutellaria baicalensis extract by IPSE chip to obtain aglycones and glycosides: (**A**) IPSE chip. (Aa) IPSE chip when filled with dye mixture (indigo and Sudan red); (Ab) IPSE chip when using dichloromethane-butyl acetate (2:8) to separate the dye mixture. (**B**) HPLC profiles: baicalin (peak 11), wogonoside (peak 12), baicalein (peak 13), wogonin (peak 14). The image from [10], Copyright 2021, MDPI.

Separation and purification can be improved by combining microfluidics with LLE or induced phase separation extraction (IPSE). IPSE is a modification of LLE, in which organic solvents are separated from water in seconds through the addition of salt or hydrophobic solvents or cooling of the solution to a temperature below zero. Ideally, the components can move quickly to more affinity solvents due to differences in substance properties. By using the separation principle of IPSE, a solution containing the target product can be obtained quickly. However, there is still a need for automation due to the complex operation of IPSE, and microfluidics can help reduce the complexity of the operation. For example, a valuable experiment was conducted in 2021 to develop an IPSE chip using microfluidic technology. The addition of a hydrophobic inducer resulted in the separation of the sample solution of acetonitrile–water into separate organic and aqueous phases. The adjustment in the acid/base properties of the sample solution affects the distribution of the compounds. In this experiment, aglycones and glycosides were successfully separated from *Scutellaria baicalensis* extract, as shown in Figure 11. Sample and solvent usage were whittled down. The sample pretreatment time was shortened, the extraction efficiency was higher than that of the LLE chip, and the formation of emulsions was prevented. More importantly, the operation is far less tedious compared to macro-LLE or IPSE [10].

In the above section, we detailed some explorations on two-phase microfluidic chips, three-phase microfluidic chips, continuous laminar flow microfluidic chips, and IPSE microfluidic chips, all with invariably excellent results over macroscopic methods. In addition to the aforementioned microfluidic experiments for extracting the active ingredients of herbs, there are many more explorations in this area (Table 2).

#### 3.1.2. Quality Control of Drugs from Improved Formulations of Traditional Medicine

The drugs obtained by modifying formulations of traditional medicines require quality control to ensure the safety, efficacy, and consistency of the drug. This is because the raw material growth location, conditions, different production methods, and differences in the drug production process may result in the drug having a different chemical composition and therapeutic effect [2]. Another reason is to prevent the addition of additives that are harmful to humans, such as formaldehyde, during the preparation process [6]. The quality control of pharmaceuticals is still mainly performed using methods that detect certain specific components in the sample, but the complexity of the sample composition may affect the detection process, leading to unreliable conclusions. Therefore, extracting the target molecule from a complex sample before performing the assay can greatly improve the accuracy of the assay results. Microfluidics can provide a very beneficial contribution to the extraction process. In the next section, we will present some experimental designs of microfluidic techniques applied to separation and purification methods. These designs enable the quality assessment of new drugs developed by improving the formulation of traditional pharmaceuticals.

**Figure 12:**
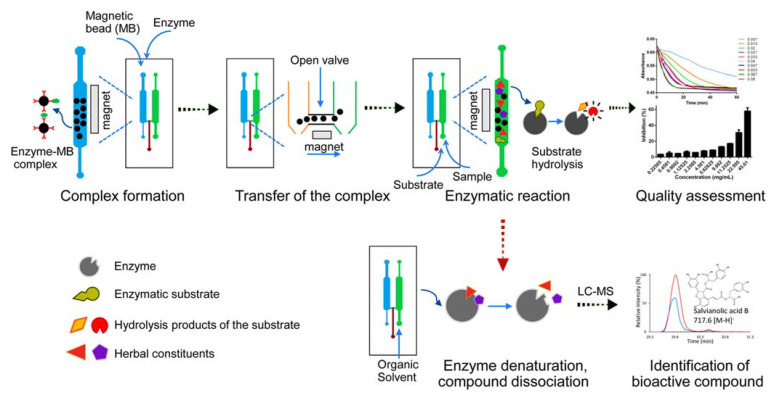
Schematic diagram of biopharmaceutical quality assessment and screening on a Microfluidic chip. The image from [2], Copyright 2017, Scientific Reports.

Ling Lin et al. developed a microfluidic system for the extraction and detection of catechins from green tea by combining solid-phase microextraction and chemiluminescence. Specifically, it is divided into two steps. Firstly, the monolithic column is integrated into the microfluidic chip to achieve the extraction of catechins from green tea. Then, the catechins react with potassium permanganate to produce chemiluminescence. The reason for using the monolithic column as the extraction material is its good permeability, convenience, and enrichment, while the use of microfluidics enables the automation, integration, and miniaturization of the analysis, which has tremendous benefits in terms of reduced reagent usage and time reduction. Finally, since the reaction of catechins with potassium permanganate can produce chemiluminescence directly, additional manipulation and the influence of external light sources can be avoided, and the effects caused by stray light and light source fluctuations can be excluded. In their analysis, the linear range was 5.0 × 10 ^−9^–1.0 × 10 ^−6^ M, and the limit of detection was 1.0 × 10 ^−9^ M; the recoveries were able to reach 90%–110%; in addition, the relative standard deviation (RSD) was found to be 4.8% by parallel measurement of 10 samples with 1.0 × 10^−8^ M catechins. These data show that the system has satisfactory linearity, sensitivity, recovery, and precision. The system eliminated the tedious elution step and had high sensitivity, low consumption, and reusability, thus being an inexpensive and high productivity means of testing the quality of green tea [61]. Often, the therapeutic activity of conventional medicine is due to the synergistic and simultaneous action of several chemicals; therefore, confirming only the content of a single ingredient may not be realistic [1]. Since the determination of single chemical composition is not sufficient, the determination of multiple chemical compositions may be one of the favorable methods for increasing the convincing power. Attempts to control the quality of drugs by testing multiple ingredients were made more than a decade ago. A flow injection (FI)-microfluidic capillary electrophoresis (CE) microfluidic chip, suitable for the determination of the main components in pharmaceutical preparations, was proposed in 2003. This experiment determined the content of four alkaloids (ALP, SRI, MT and OMT) in two marketed drugs and proved the efficiency, reproducibility, and applicability of the FI-CE microfluidic chip. It is a promising method for both drug quality control and routine analysis and monitoring [62]. In addition, Tsung-Ting Shih et al. combined microfluidic electrophoresis and electrochemical detection with a simple derivatization method for the determination of five representative components of hesperidin, including Pinellia ternata guanosine, methionine, glycine, 3,4-dihydroxybenzaldehyde, and homogentisic acid. The improved efficiency and reduced sample and reagent consumption provided a promising idea for the efficient analysis of complex components [63]. Additionally, in 2017, Mad et al. provided an interesting experimental design for the completed biological pharmaceuticals, as shown in Figure 12. Ahmad et al. evaluated the quality of QiShenYiQi pills (QSYQ) using microfluorescence and assessed the biological consistency by enzyme inhibition of thrombin and angiotensin-converting enzyme (ACE) as quality biomarkers. QSYQ is a pharmaceutical product containing plants such as Astragalus, Salvia, Panax ginseng, and Dalbergia odorifera. The complexity of the constituents led to the undesirability of the strategy of quality assessment based on the presence or absence of one or several molecules within the drug. They innovated the approach of judging the effectiveness of pharmaceuticals based on the changes in the biomarkers. Three functions, namely, enzyme–MB complex formation, and enzyme reaction and screening, were combined in a multifunctional chip. Then, enzyme inhibition (percentage) was used as an indicator of drug quality; and the HPLC system combined with a variable wavelength detector (VWD) was used for a fingerprint analysis. After studying five batches of QSYQ, differences in the effects on thrombin and ACE were found between batches, demonstrating the validity of the method. More positively, they further explored the linearity, reproducibility, and reliability of this strategy. The results showed an r2 of 0.9988 and a repeatability of less than 15% for thrombin; and an r2 of 0.9810 and a repeatability of the same less than 15% for ACE. The reliability of the strategy was then demonstrated by using AEBSF-HCl and captopril as positive controls, measuring their IC50, and comparing them with literature data. Moreover, in this study, not only the quality assessment was achieved, but also tthe discovery of molecules that actually have a real therapeutic effect. Tannins, B hydrochloride, tannic acid C, and rosmarinic acid showed potent inhibition of thrombin, while tannins inhibited ACE [2].

The above examples show the importance of efficacy and quality assessment of new drugs developed by improving traditional drug formulations. However, it is worth noting that the hazards in additives are also of great concern in quality control. A well-known example is “formaldehyde”. Excessive absorption of formaldehyde can lead to adverse reactions such as headaches, abdominal pain, vomiting, and breathing difficulties [6]. Hence, ensuring that the level of formaldehyde in pharmaceuticals is within the safe range is important for human health. However, as previously shown, the separation and enrichment step is important for eliminating the influence of other compounds and accurately detecting formaldehyde in the sample. The development of microfluidics has given new ideas on how to determine the concentration of formaldehyde. Lung-Ming Fu et al. determined the concentration of formaldehyde by measuring the fluorescence intensity signal of the reactants in the collection chamber. The principle is that the samples are first mixed with a fluorescent derivatization reagent (Fluoral-P); then, heated (30 °C) for a period of time (4 min); and finally, the formaldehyde concentration is determined by the fluorescence intensity under a microscope in a laser-induced fluorescence (LIF) detection system. They used the detection system to test standard samples with known concentrations and compared the results with the traditional UV/visible absorption spectroscopy method to demonstrate the reliability of the system. The fast and easy nature of this detection system provides great convenience for the detection of formaldehyde content. In addition, by measuring the formaldehyde concentration of ten commercial Chinese medicines, the practical application value of the device was assessed [6]. The above experimental explorations are all attempts to validate that the chemical composition and therapeutic effects of the final product are influenced by differences in geographic origin, growing conditions, agricultural production methods and manufacturing processes, and the addition of additives harmful to humans during the preparation process by combining assay methods with microfluidic chip technology. These experimental studies have a very positive value and can help the use of drugs in practice, as well as provide good ideas for other scholars’ research. When drugs enter the body, the real-time monitoring of changes in the concentration of certain dangerous ingredients to prevent adverse reactions is also important for human safety considerations.

#### 3.1.3. Real-time Monitoring of Drug Concentrations in Body Fluids

Quantitative analysis is quite challenging in situations where a wide variety of contaminants are present in urine, saliva, plasma, and blood samples, and the target drugs are frequently present at low concentrations [7]. However, drug abuse-related adverse events are frequently reported. Some TCM substances believed to be potentially hazardous are listed in Table 3, emphasizing the need for efficient and timely detection strategies.

To perform a rapid, convenient, and quantitative analysis of the target drug, it is necessary to minimize matrix effects, eliminate contaminants, preconcentrate analytes, and collect samples in a form that is compatible with the analytes in the instrument. So, in clinical application, the price and operation of such technologies should not be excessively high and tedious, respectively. Microfluidic technology has the benefits of low cost, high extraction efficiency, and easier implementation of automation, integration, and parallelism, allowing it to meet these needs [7]. Ephedrine (EPH) (2-methylamino-1-phenylpropan-1-OL), extracted from ephedra, is an example of a drug whose concentration must be strictly regulated. It is a central nervous system sympatholytic stimulant that prevents hypotension and stimulates adrenergic receptors, thereby increasing blood pressure and heart rate. However, misuse of the drug can result in adverse cardiovascular reactions and even death. Mahdoo Baharfar et al. created a microfluidic device that combines the benefits of electromembrane extraction and microfluidic devices, thus specifying an electromembrane extraction (EME) on a chip for extraction and preconcentration of ephedrine from human urine and plasma samples. Utilizing central composite design (CCD) technology, the effective parameters of the extraction process were optimized: low sample volume requirements, adequate sensitivity, and low limit of detection (LOD). By increasing the surface-to-volume ratio and creating a uniform electric field along the entire length of the channel, the extraction efficiency was enhanced. In addition, the small distances between the electrodes allow a low voltage to generate a large electric field. These attributes have influenced the design of portable analytical devices [37].

Jin Sheng et al. also invented a hybrid quartz capillary/polymethylmethacrylate microfluidic device (HMD) for the rapid and sensitive detection of psychotropic drugs in blood by UV light. This takes advantage of the fact that psychotropic substances can absorb UV light at 200 nm, and takes advantage of the high efficiency, reduced reagent loss, and reduced time of the microfluidic device (MED). Specifically, fused silica capillaries, which tend to form breaks at relatively low sampling voltages, can then provide separation channels and a window without cladding for these drugs. It is also worth advocating that such an assay device can be reused after electrophoresis buffer rinsing, which greatly saves costs. The results show that baseline separations for barbiturates (phenobarbital and barbiturates), benzodiazepines (nitrazepam, clonazepam, chlordiazepoxide, alprazolam, and diazepam), and a tricyclic antidepressant (amitriptyline) were achieved within 200 s with 3.8 × 10^5^ plates m^−1^ at the maximum separation efficiency. The difference is that the conventional methods of capillary electrophoresis and high-performance liquid chromatography take 12 and 15 min, respectively. Coupled with the compact and convenient nature of the device, the design and popularity of the device are of great significance if they can meet the detection needs. Moreover, the detection limits of the device for the eight psychotropic substances were as low as 27 ng/mL (phenobarbital) and as high as 67 ng/mL (chlordiazepoxide) in plasma samples spiked with standard levels of psychotropic substances. Therefore, the device is fully capable of reaching the clinically effective therapeutic concentration of 100 ng/mL. In contrast to the lowest concentration of 500 ng/mL that is harmful to humans, the linear range of the other seven drugs can reach the upper limit of detection of 1000.0 g/mL, except for chlordiazepoxide, which has an upper limit of detection of 2000.0 g/mL. Therefore, their sensitivity and linear range fully meet the needs of the assay. So, this study provides a promising method for the preliminary screening of psychotropic drugs with high resolution, rapid separation, and low expense. Of course, it would be preferable to have a further improved strategy to be able to determine directly in the device exactly which psychotropic drug is in the sample [64].

A microfluidic device involving the use of a bio-nanopore consisting of a biological nanopore bound to a DNA aptamer has also been prepared. This chip enables on-the-spot drug detection and rapid detection of small molecules [29], even detecting cocaine at concentrations as low as 300 ng mL^−1^ in 60 s. Another microfluidic device containing a mid-infrared single-mode strip waveguide that can be used for cocaine detection has also been prepared. However, this device has a minimum detection concentration of 100 mg/mL, which is a significant shortfall compared to other assays that can detect nanogram orders of magnitude per milliliter [65].

We have highlighted several recent examples of combining microfluidic chip technology with monitoring methods to demonstrate the superiority and universality of microfluidic chip systems. We believe this can provide better ideas for more experimental design solutions, and we look forward to more applications of microfluidic chip technology.

### 3.2. Screening of Traditional Medicine Active Ingredients

Regardless of the approaches to drug development from traditional medicine, drug screening is a mandatory part of the process. However, traditional in vitro cellular models and in vivo animal models are not suitable options. Based on cell colorimetric assays, microtiter plate-based cytotoxicity assays, such as MTT bromide (3-[dimethylthiazol-2-yl]-2,5-diphenyltetrazolium) assay, are widely used as in vitro cellular models for drug screening. However, the conventional test is time-consuming and involves many herbal components. In addition, since the test uses a colorimetric method, antioxidants and colorants may alter their reliability and sensitivity. This, coupled with the inability to replicate changes in the in vivo microenvironment, has led to unpredictable changes induced by drugs in vivo. In vitro animal models likewise have a non-negligible impact on the drug screening process. The high cost of animal models, the difficulty in handling them, the difficulty of genetic and physiological differences in humans, and the inevitable ethical issues pose obstacles to drug development [14]. In addition to the problems mentioned above, because the therapeutic activity of conventional medicine is often due to the synergistic and combined interaction of multiple molecules, obtaining truly valuable findings is non-negotiable; this often requires the testing of large chemical combinations and relies on data at multiple qualitative and quantitative levels [15]. These factors have shown the need to look for new drug screening methods to advance the drug development enterprise. Microfluidic platforms are an attractive option for high-throughput analysis during drug development. This is because a multifunctional integrated system based on microfluidic chips offers low cost, high throughput, speed, sensitivity, specificity, and affordability. In addition, it has the great advantage of mimicking the human environment. Therefore, the application of microfluidic chip technology can effectively solve the problems of complexity and ambiguity of the mechanism of action of traditional medicines and advance the process of drug development [16,66]. In this section, classical examples are given to introduce improved variations in experimental design in this area, with the expectation of providing good ideas for drug development from traditional medicine.

**Figure 13:**
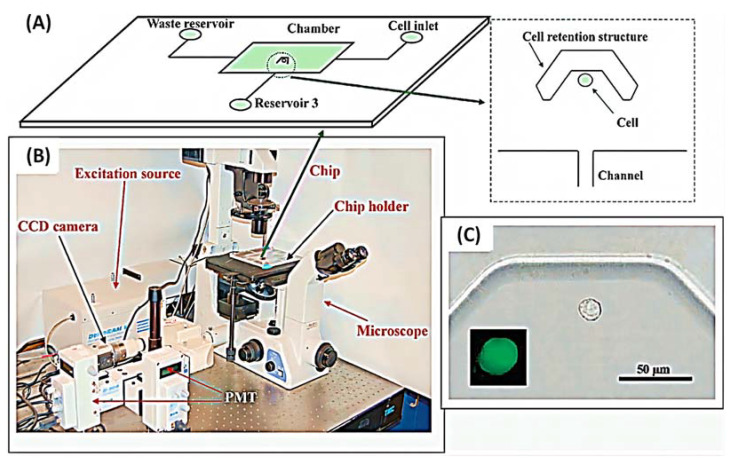
Schematic diagram of the layout and instrument setup of the Microfluidic chip: (**A**) Schematic diagram of the Microfluidic chip. (**B**) Schematic diagram of the instrument setup. (**C**) Single cells were fixed with a scale bar of 50 mm. The inset shows the fluorescence image of a RAW cell (7 mm in diameter) after stimulation with 10 mg/mL of ionomycin. The image from [14], Copyright 2009, The Royal Society of Chemistry.

Microfluidic chips that detect pharmacological effects by measuring changes in Ca^2+^ were introduced in 2008. Ca^2+^ elevation is a well-known early cytotoxic event. The use of v-shaped microfluidic chips to pick single cells not only reduces the cost of reagents and the requirement for cells but also discloses several phenomena that are not visible in the bulk analysis due to cell heterogeneity. More importantly, activities that would have taken days in a traditional experiment were completed in hours, with comparable outcomes. The layout of the microfluidic chip and the setup of the instrument are shown in Figure 13 [14]. In this experiment, the inlet is on the right side and the outlet is on the left side. The cells will be caught due to the v-shaped structure. Finally, the results were observed by fluorescence microscopy. Additionally, due to the advantages of microfluidic technology in real-time single-cell analysis and repeatable medium exchange, the effect of thymus plant extracts on the morphology of individual breast cancer cells could be monitored in real-time by precisely controlling the flow of drugs through the culture chamber in a study by MohdRidzuanAhmad. The advantage is that observing changes in cell shape rather than detecting changes in Ca^2+^ is more convincing. In this device, the cells are captured in a fixed area when a certain flow rate of cell suspension flows through due to the limitations of the microchannel. The cells are then incubated for a certain period of time and then injected with a solution containing a different concentration of the potential drug, and after 3 h of observation of the cell changes, the drug with therapeutic effects can be screened. The experimental result shows that high concentration thymus (560 g/mL) has a significant impact on the cell membrane, but low concentration thymus (15 g/mL) has a negligible impact [17]. This operation is not meaningless, because setting a drug concentration gradient on a microfluidic chip is important for determining the appropriate dose impact and providing guidelines for use in clinical medication. However, medication combinations are frequently employed in clinical settings because single drugs are susceptible to resistance and may harm healthy cells. Combining medications has the advantages of preventing drug resistance, lowering toxicity, and enhancing therapeutic efficacy.

**Figure 14:**
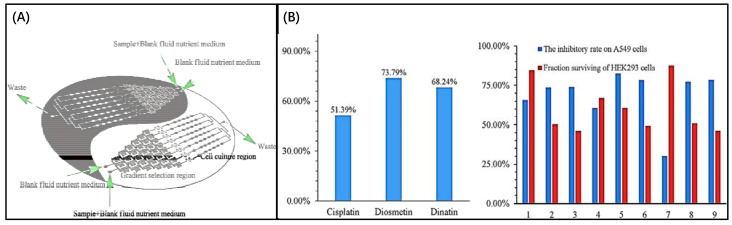
Schematic representation of cisplatin, dinatin, diosmetin activity, and toxicity screening by Microfluidic chip: (**A**) Schematic diagram of the chip. (**B**) The calculated survival rate of cisplatin, dinatin, and diosmetin to HEK293 cell, along with the results of each compatibility group. The image from [16], Copyright 2019, Elsevier.

**Figure 15:**
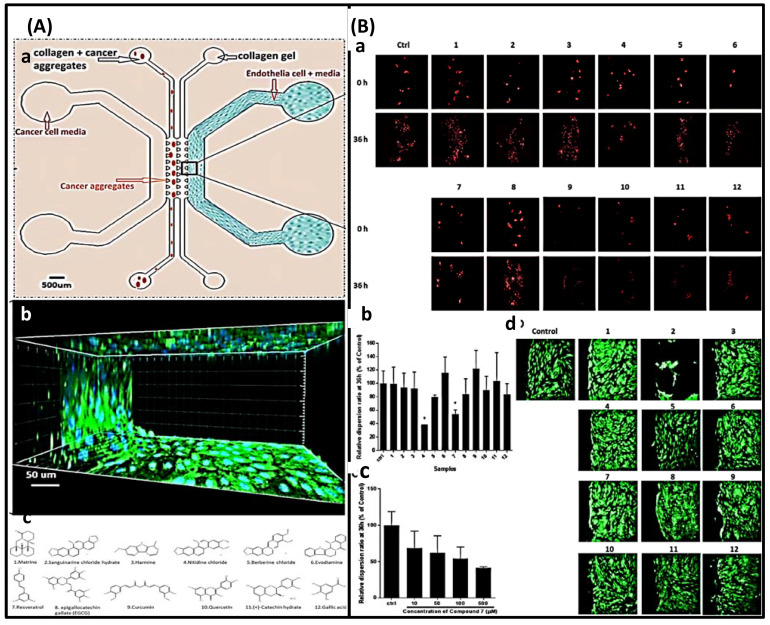
(**Aa**) Schematic diagram of the microfluidic system. (**Ab**) Verification of the integrity of the endothelial cell monolayer between collagen and HUVEC channels by immunofluorescence staining for VE-cadherin (green in the image). (**Ac**) List of 12 compounds tested in this study. (**Ba**) Images of A549 cancer spheroids before and after 36 h of treatment with various drugs. (**Bb**) Relative dispersion rates of spheroids in each sample group. (**Bc**) Relative dispersion rates of spheroids under the effect of different concentrations of compound 7. (**Bd**) Representative images of HUVECs prearranged along the channel (Calcein-AM staining in green) after 36 h of treatment with various chemical drugs, with compound 2 causing significant damage to the endothelium. The image from [18], Copyright 2014, American Chemical Society.

In 2019, Hechen Wang et al. invented a multifunctional integrated microfluidic biochip device with two “Yin and Yang” modules that placed healthy cells and disease cells separately (detecting drug toxicity and activity at the same time), as shown in Figure 14A. Similarly, the cell solution is injected into this microfluidic device and the microchannels are able to immobilize the cells at a certain flow rate. Notably, there are two inlets at the top of the device, which can produce eight concentrations when entering different solutions due to the special structure. After a certain time of drug action, fluorescent staining is performed and the situation is recorded by fluorescent microscopy, and finally, the apoptosis and necrosis rates of the cells are calculated using Image-Pro Plus software. In addition, the advantage of a concentration gradient that can detect optimal medication compatibility can be configured to achieve optimum efficacy and least toxicity. They evaluated the therapy-toxicity of cisplatin (a chemotherapy agent with kidney toxicity), dinatin, and diosmetin (natural products of Traditional Chinese Medicine derived from the total flavonoids of *Cirsium setosum* (Wild)) by estimating apoptosis and necrosis rates as shown in Figure 14B. The results of the microarray and 96-well plate assays showed a good correlation and no significant changes in both sets of numbers, indicating that the microarray has a high degree of accuracy in the detection of activity and toxicity. In addition, the efficacy and toxicity of different compatibility groups were tested. This investigation revealed that various groups had distinct inhibition rates on A549 cells and varying toxicity on HEK293 cells, suggesting that dosage compatibility can minimize the toxicity of cisplatin and enhance its inhibitory effect on A549 cells. The study by Hechen Wang et al. offers new techniques for predicting toxicity, screening medications, and investigating drug action mechanisms, as well as exploring novel concepts for tumor therapy, chemotherapeutic drugs, and the combination of active compounds in Traditional Chinese medicine [16]. In addition to single-cell and 2D microfluidics, which have garnered considerable attention, it is impossible to disregard 3D microfluidics that can imitate the human microenvironment. For example, 3D microfluidics is used in the creation of new antimetastatic medications derived from natural ingredients. Using a 3D microfluidic device that co-cultured endothelial cells in 3D collagen gels and cancer cells formed as 3D spheroids, Yiming Niu et al. verified the antimetastatic effects of 12 natural compounds. A schematic of the engineered microfluidic system used for composite screening is shown in Figure 15Aa. The channels are supplemented with a collagen solution, which supports the adhesion of endothelial cells after gelation. The human lung cancer cell line, A549, is cultured to form spheroids, which are collected and mixed in a pre-optimized collagen buffer. Subsequently, they are transferred into the indicated channels, and the average distance between the endothelial layer and the cancer spheroids determines the distance that allows sufficient cell–cell communication in a typical tumor microenvironment. Twelve natural compounds were tested in this system (Figure 15Ac). The secretion of human umbilical vein endothelial cells (HUVECs) can diffuse into the collagen matrix along with drug molecules, and a photograph of a sample of fluorescently stained cells in this device is shown in Figure 15Ab. Figure 15Ba,Bb show that compounds 4 and 7 of these 12 natural compounds significantly inhibited metastasis. We further tested compound 7 at 10, 50, 100, and 500 μM and found that its dose dependence between 10 and 100 μM was less pronounced than that between 100 and 500 μM (Figure 15Bc). Meanwhile, in the endothelial channel, compound 2 was the only compound that significantly disrupted the endothelial cell layer, as shown in Figure 15Bd, based on cell morphology observation and live cell staining. We believe that compound 2 has a specific inhibitory effect on endothelial cells and warrants evaluation of its anti-angiogenic potential. Except for these three molecules, all other samples showed no inhibition of spheroid dispersion or significant effects on endothelial cells [18].

**Figure 16:**
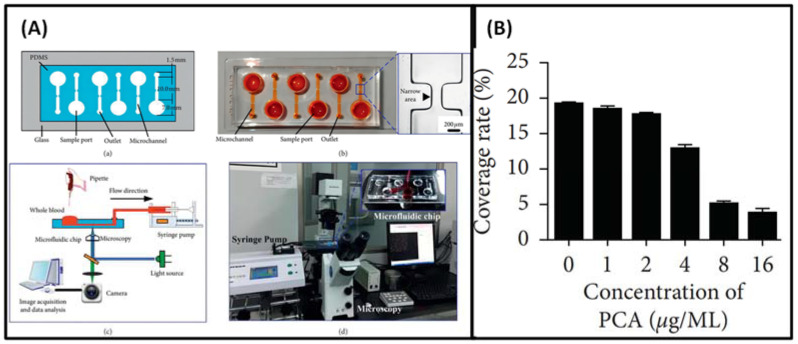
(**Aa**) Schematic diagram of the microfluidic chip. (**Ab**) Physical diagram of the microfluidic chip. (**Ac**) Schematic diagram of the working principle of the analyzed system. (**Ad**) Photograph of the analytical system. (**B**) Histogram of platelet surface coverage for different concentrations of pro-catechin. The image from [22], Copyright 2021, Hindawi.

**Figure 17:**
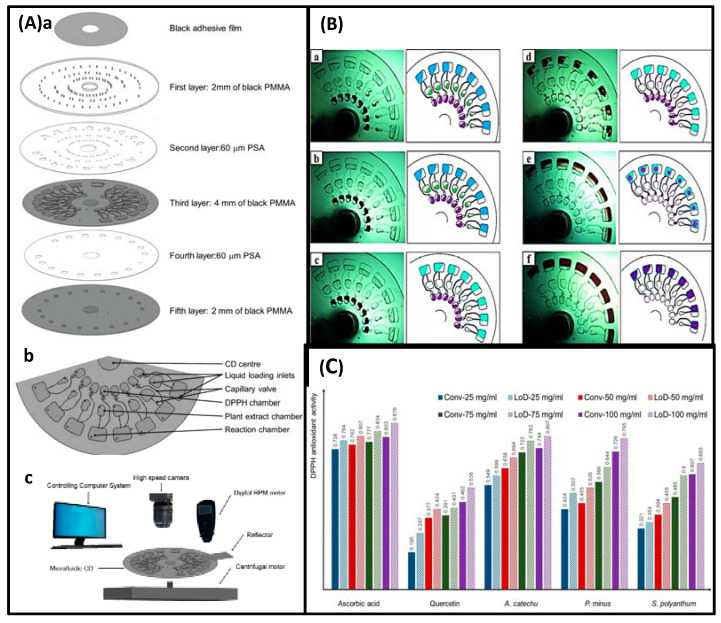
(**Aa**) Structural view of the Microfluidic compact disc (CD). (**Ab**) Top view of the third layer of the antioxidant microfluidic chip. (**Ac**) The whole experimental setup. (**B**) Image and schematic diagram of the whole process on CD. (**Ba**) Initial of the experiment, speed is 0. (**Bb**) The plant extract starts to flow into the capillary valve, speed increases from 0 to 300 rpm. (**Bc**) The plant extract chamber is emptied, speed 300 rpm. (**Bd**) The DPPH solution flows from its reaction chamber to the capillary valve, speed increases slowly from 300 to 800 rpm. (**Be**) The DPPH reaction chamber is emptied, speed 800 rpm. (**Bf**) All solutions were properly mixed in the reaction chamber at a speed of 1400 rpm. (**C**) Comparison of the conventional and LoD methods at different antioxidant activities. The image from [67], Copyright 2018, MDPI.

**Figure 18:**
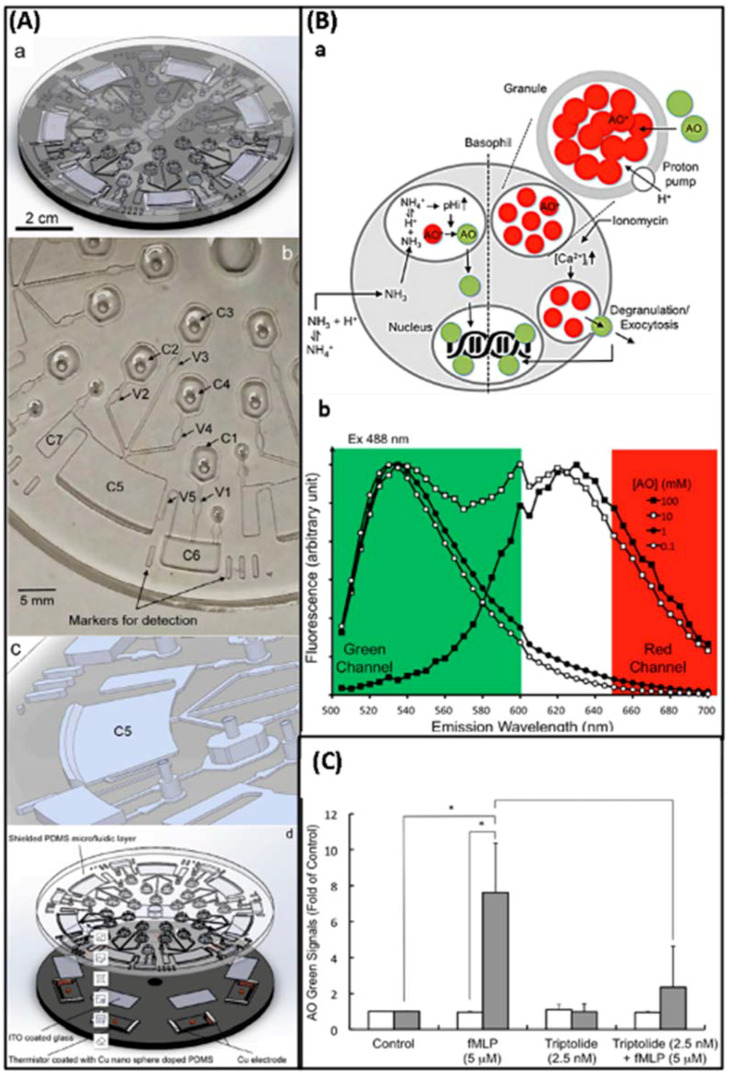
**(Aa)** Schematic diagram of the LOAD layout. **(Ab)** Chambers, channels, valves and siphons for testing allergenicity in one unit. **(Ac)** A zoom-in diagram showing the flange in C5. **(Ad)** Assembly of the integrated microfluidic layer. **(Ba)** Schematic diagram of AO as a degranulation reporter. **(Bb)** Red-shift phenomenon. **(C)** Triptolide suppressed the fMLP-mediated AO release in KU-812 cell. The image from [68], Copyright 2016, MDPI.

Microfluidic technology is employed not only for the screening and combination of anti-cancer medications but also for screening natural drugs for anti-platelet aggregation, antioxidant capacity, allergy suppression, and nerve damage relief effects. For instance, 3D microfluidics was used in a study that investigated the inhibitory effect of protocatechuic acid on shear-induced platelet aggregation. Changes in fluid shear rate gradients, which can cause platelet aggregation, can be simulated using 3D microfluidics. This only requires the design of a microchannel structure similar in size and structure to the microvasculature and then the control of the flow rate to simulate the in vivo microvasculature. Moreover, fluorescence microscopy was used to study platelet adhesion and aggregation, and the inhibitory impact of protocatechin on shear rate gradient-induced platelet aggregation was observed in real-time. In the experimental concentration range of 0–8 mol/mL, it was found that protocatechuic acid inhibited platelet aggregation in a concentration-dependent manner, as shown in Figure 16 [22]. In measuring the antioxidant capacity of plants, Nurhaslina Abd Rahman et al. developed the lab-on-a-disc (LoD) method, which incorporates the DPPH (2,2-diphenyl-1-picrylhydrazyl) antioxidant activity test on a microfluidic compact disc (CD). This microfluidic CD consists of five layers: the first layer is a transparent Polymethyl Methacrylate (PMMA) disc (with injection/exhaust holes) covered by a black adhesive film except for the reactor chamber; the second layer is pressure-sensitive adhesive (PSA); the third layer is a PMMA disc (black) engraved with microfluidic channels and chamber features; the fourth layer is PSA; and the fifth layer is black PMMA. The device actually controls the flow and mixing of liquids by placing different reactant solutions in different chambers, using changes in rotational speed and capillary passive valves. As well as reading the absorbance of antioxidant activity after a certain time of reaction to determine its antioxidant effect. Using plant extracts of ascorbic acid, quercetin, *Areca catechu*, *Polygonum minus*, and *Syzygium Polyanthum*, the LoD method was compared to the conventional method. Repeated ANOVAs were performed with IBM SPSS statistical software, and the results showed that the LoD method is efficient and straightforward for the determination of antioxidants, as shown in Figure 17 [67]. In the same vein, Ho Chin Kwok et al. also used this reliance on telecentric forces to move agents and cells around in an experimental design. However, they combined this with the indium tin oxide (ITO) microheater pathway, they found a traditional drug ingredient (tretinoin) that effectively inhibits allergic reactions, as shown in Figure 18 [68]. Furthermore, in measuring antioxidant capacity, there is also a microfluidic technique paired with a novel instrument for peroxyoxalate chemiluminescence (PO-CL). The principle of this is that since the production of CL requires the help of hydrogen peroxide, when the antioxidant removes the hydrogen peroxide, the CL is inhibited and the light production is reduced. By fixing the amount of peroxynitrite and hydrogen peroxide, the activity of the antioxidant can be determined by the reduction in light. Using 9,10-bis- (phenylethynyl)anthracene (BPEA) as a fluorescent probe and hydrogen peroxide as an oxidant in this experiment, carotene was determined to be the most effective hydrogen peroxide scavenger among the tested botanical antioxidants (SAHP = 3.27 × 10^−3^ mol^−1^ L), followed by -tocopherol Phenol (2.36 × 10^−3^ mol^−1^). In this experiment, the infusion of antioxidants was regulated by an injection valve, which increased detection sensitivity and reaction time. By optimizing the on-chip injection of antioxidants and employing smaller and deeper microchannels, sensitivity and throughput can be enhanced further. This compact automated instrument is incredibly useful for assessing the antioxidant capacity of inexpensive plant-based foods and pharmaceutical supplements on-site [59]. The effect of bidentate polypeptide k (ABPPk) on the survival, growth, and axonal regeneration of spinal cord motor neurons was one of the tests conducted for the screening of natural medicines for the treatment of nerve injury. A co-culture model of motor neurons and skeletal muscle cells found that ABPPk stimulated the maturation and formation of neuromuscular connections. This study offers a foundation for the prospective utilization of ABPPk in the development of therapeutic strategies for nerve injury diseases [23].

In addition to these experimental designs, several studies combine microfluidics with bioinformatics to conduct further investigations into the mechanisms of drug action. Attempts have been made to provide a theoretical basis for improving drug quality, enhancing drug efficacy, and preventing adverse effects.

In 2017, two teams employed microfluidic technology to uncover active components in natural substances and investigate their mechanism of action to combat lung cancer. Even though FAN Jia-xin et al. concluded that Nepeta must have a certain inhibitory impact on lung cancer cells based on a past literature study and pharmacodynamic trials, the pharmacological mechanism remains unclear. They used a microvalve-structured double-layer composite chip combined with ultra-high performance liquid chromatography–mass spectrometry to analyze the ethanol extract of Nepeta mustard based on the chromatographic retention time, molecular fragmentation peak, accurate molecular weight, database, and control. Comparing the product information and analyzing and identifying the chemical makeup, five components play a significant role in suppressing the proliferation of lung cancer cells, as demonstrated by the results of the experiments [50]. However, this study did not explore the pharmacological and pharmacodynamic aspects in sufficient depth. In contrast, a study by Jiaxin Fan et al., targeting flavonoids (TFS), provided valuable insights. In their investigation, they utilized a microfluidic chip combined with flow cytometry to examine the effect of TFS isolated from Nepeta mustard on the human lung cancer cell line A549. To investigate the molecular mechanism of a portion of the PI3K-AKT pathway, quantitative real-time PCR (qRT-PCR) and Western blot were employed to assess the mRNA and protein expression of microRNA-126 (miR-126), VEGF, PI3K, and PTEN. The findings indicated that TFS could inhibit the expression of miR-126, modulate the PI3K-AKT signaling pathway, and exert anticancer properties [4].

There is a clear example of the close relationship between energy metabolism and inflammation in activated microglia. In addition, *Rhodiola* contains sorbide (Sal), which inhibits microglial hypoxia-induced inflammation (HI). However, it is unknown if sorbide (Sal) inhibits microglial hypoxia-induced inflammation (HI) by altering microglial energy metabolism. A new cell microfluidic chip mass spectrometry (CM-MS) system has enabled real-time monitoring of the effects of drug metabolites on cells to investigate the changes in metabolic processes during hypoxia [20]. In the meantime, they measured the level of HI-related factors to confirm the metabolic mechanism of hypoxic BV2 cells. The correlation between DFO-induced BV2 cell HI and energy metabolism was revealed by the integration of detected results. In addition, the administration of Sal could effectively reverse this transformation, and two Sal metabolites were identified: tyrosol and 4-hydroxyphenylacetic acid. This experiment reveals the mechanism by which Sal mediates energy metabolism mechanisms to reduce HI injury in BV2 microglia by promoting glycolysis to OXPHOS under hypoxic conditions. It paves the way for real-time, online, dynamic monitoring of the energy metabolism mechanism of drug effects on cells and provides a superior screening strategy for natural drug candidates for the treatment of brain diseases [20]. NijiaWang et al. designed an experiment to investigate the mechanism for TTS therapy in cervical cancer. In this experimental design, numerous techniques were combined, such as 3D microfusion, microfluidic chip, flow cytometry, mass spectrometry coupling, and bioinformatics. The results demonstrate that TTS first stops the transformation of cells in the G0/G1 stage to the S and G2/M stages. Subsequently, gene and protein synthesis was inhibited. Finally, it reached the goal of blocking cell proliferation and inducing apoptosis. Consequently, the experiment confirmed the effect of tannin on the associated genes and proteins [19].

All these examples are innovative experiments of microfluidic chip technology in drug screening, and they provide ideas in the process of developing a qualified drug that can be truly applied, which makes an important contribution to human health. We expect more exploration and thinking in this field through microfluidic chip technology and believe that more valuable drugs will be introduced in clinical practice.

In this section, we present some examples of research in the drug screening step of the drug development process through traditional medicine. All of them demonstrate the great potential value of this strategy. Moreover, in these studies, whether it is a strategy to probe whether a drug is effective through changes related to cell contents or changes in cell morphology; whether it is a single drug assay or a combination drug protocol; whether it is screening drugs at the 2D or 3D level; whether it is a drug for which the type of disease or experimental design is used in conjunction with whichever assay system, microfluidic chips have demonstrated great adaptability and powerful support for the drug development business [69,70].

## 4. Conclusions

In this review, in addition to briefly describing the role played by microfluidics throughout the classical drug development strategies (synthesis, screening, delivery, and vector manufacturing), we focus on the help it provides in drug development strategies from traditional medicine. The advantages of microfluidics are as follows: firstly, the ability to make traditional extraction methods more efficient, environmentally friendly, automated, and miniaturized; and secondly, the ability to make drug screening methods fast, high throughput, low cost, reliable, sensitive, and to replicate the human in vivo environment. Therefore, microfluidics plays a superb role in drug development strategies for traditional medicine in extracting target molecules from complex samples and screening the active ingredients of traditional drugs.

Microfluidics is widely used in classical drug development strategies. In drug synthesis, there are two pathways, microchannel reactor and droplet microreactor. In drug delivery, microfluidic devices can also be used as pumps to provide power. Microfluidics also plays a role in the manufacture of drug carriers. Especially in drug screening, it provides various types of options including single cell, 2D, 3D, organ, and human. Two-dimensional microfluidic chips have initially met the requirements of determining whether a drug is effective; on single-cell microfluidic chips, the problem of cell heterogeneity is solved. Three-dimensional microfluidic chips can simulate the human in vivo environment; further development of organ chips and human chips can provide deeper information on drug therapy. However, in addition to being alert to non-Newtonian flow control and by-products in drug synthesis, encapsulation materials in drug delivery also need to be carefully selected. In addition, although there has been a lot of exploration on organ-on-a-chip and human-on-a-chip, more development is needed because simplified models still cannot replicate the full range of human pathophysiological processes. In particular, the drug screening process requires high throughput and the metabolism of drugs usually requires multiple organ actions, so a large amount of data support is needed to design models that are drug metabolism oriented and efficient [26].

Synthetic drugs obtained through classical drug development strategies are not only costly and time consuming, but their side effects are also a cause for concern. The effectiveness of traditional medicine has been proven for a long time, and obtaining new drugs from this direction can significantly save time and effort, and may even address concerns about side effects. Although there are a number of obstacles to this strategy, as mentioned earlier, it has been successfully implemented due to the characteristics of microfluidic technology. However, there are six aspects worthy of attention.

First, although the combination with microfluidics can reduce the environmental damage compared to macroscopic extraction methods, it does not mean that the problem of toxic solvent use and waste emissions is completely solved. Second, limited by the microfluidic device, although the efficiency is high, the actual flux is not high. But fortunately, the problem of low throughput can be solved by parallelizing multiple microfluidic modules in parallel [5]. Third, unlike single-active-ingredient drugs, new drugs developed by direct modification of traditional drug formulations require not only quality assessment, but also vigilance against hazardous additives due to their vulnerability to the preparation process. In addition, these formulated drugs may contain certain compounds with potential toxicity. Although microfluidics plays an important role in quality assessment (chemical testing), determining the effectiveness of a drug is not accurate due to the complexity and ambiguity of the active ingredients. Although microfluidics can help reduce detection limits when testing for additives and potentially toxic compounds, it is still limited by the assay system itself. Fourth, also in the drug screening process, there is a need to further develop new models built on microfluidic chips to replace animal experiments and human experiments. Fifth, although the technology for preparing microfluidic devices is relatively mature, more research may be needed due to the limitations of complex microchannels, resistance to solving corrosion, or other special conditions. Sixth, although the development of new drugs from traditional medicine is likely to be an effective way to break through the current drug development dilemma, classical drug development strategies cannot be dismissed in their entirety and are compatible in terms of potential drugs for chemical modification, screening, and delivery.

In general, microfluidics has an extremely important position in either strategy, driving the flourishing drug development business, and as the research progresses, the above-mentioned problems will be properly solved. Through this review, we hope to provide new ideas and references for scholars, so that the human health business will flourish.

## Figures and Tables

**Table 1:** Summary of micro-engineered organ models. The image from [51], Copyright 2011, Elsevier.

Organ	Incorporated Cell Typets	Demonstrated Organ-Specific Features
Liver	Hepatocytes	Serum protein synthesis
	Vascular endothelial cells	Bile canalicull
	Fibroblasts	Liver sinusoid
		Liver zonation
Lung	Airway epithelial cells	Airway closure and reopening
	Aiveolar epithelial cells	Small airway protein (CC10) synthesis
	Pulmonary microvascular endothelial cells	Alveolar-capillary interface
		Surfactant production
		Lung inflammation
		Extrapulmonary absorption
Kidney	Renal tubular epithelial cells	Molecular tranpor
Gut	Intestinal epithelial cells	Intestinal absorption
Bone	Osteoblasts	Lacuna canalicular netwod
	Osteocytes	
Breast	Mammary epithelial cells	Malignant tumor invasion
	Mammary fibroblasts	Cancer metastasis
	Vescular endothelial cells	
Eye	Corneal epithelial cells	Epithelial barrier function
	Vascular endothelial cells	
Brain	Neurons	Axon-glia interaction
	Astrocytes	Tumor angiogenesis
	Oligodendrocytsr	

**Table 2:** Microfluidic chips for the separation and purification of active ingredients.

Herbal Medicine	Microfluidic Chips	Specific Content	Refs
Olive	Two-phase microfluidic chip	Comparing the efficiency of two-phase chip and three-phase chip in extracting and separating non-polar and polar components with different clinical effects, the three-phase chip greatly improved the efficiency by double liquid-liquid interface area.	[5]
Ginseng	Two-phase microfluidic chip three-phase microfluidic chip continuous two-phase microfluidic chip	Comparing the extraction efficiency of two-phase chip, three-phase chip and continuous laminar flow chip for ginsenosides, it was found that the extraction efficiency of continuous laminar flow chip was higher than that of three-phase laminar flow chip, and the extraction efficiency of both chips was higher than that of two-phase chip.	[3]
Strychnos seed	Two-phase microfluidic chip three-phase microfluidic chip	Comparing the efficiency of strychnine extraction on the two-phase chip and three-phase chip, the extraction efficiency of three-phase chip was higher than that of two-phase chip. And the alkaloids (stychnine and brucine) were purified from the seed extracts using the three-phase chip.	[8]
Salvia miltiorrhiza	Two-phase microfluidic chip three-phase microfluidic chip	Comparing the efficiency of two-phase chip and three-phase chip in extracting and separating non-polar and polar components with different clinical effects, the three-phase chip greatly improved the efficiency by double liquid-liquid interface area.	[9]
Scutellaria baicalensis	IPSE two-phase microfluidic chip LLE dual-phase microfluidic chip	Comparing the efficiency of IPSE duplex chip with IPSE and LLE duplex chips for the extraction of chlorogenic acid, rutin, epigallocatechin gallate, quercetin, santonin alizarin. The results showed that the extraction efficiency of the duplex IPSE chip was higher than that of the macro-IPSE and duplex LLE. And the aglycones and glycosides present were successfully separated from Scutellaria baicalensis extract by IPSE duplex microarray.	[10]
macleaya cordata seed	Two-phase microfluidic chip three-phase microfluidic chip	Comparing the extraction efficiency of potent alkaloids (chelerythrine, sanguinarine, protopine, and allocryptopine) from Macleaya cordata seeds extract by G-quadruplex technique with that of three-phase microarray, two-phase microarray, and traditional ultrafiltration method. The three-phase chip was found to be highly efficient.	[11]
green tea (catechins)	Solid-phase microextraction microfluidic chip	A solid-phase extraction microfluidic chip was developed for the successful extraction of catechol from green tea. In combination with chemiluminescence, a reusable catechin analysis system with high sensitivity and very low reagent consumption was successfully established. The system enables online pre-concentration and detection without elution steps, while providing good stability and reusability.	[61]
bio-alkaloids (Matrine; Sophoridine; oxymatrine; aloperine)	FI-CE microfluidic chip (Flow injection -capillary electrophoresis)	Development of a FI-CE microfluidic chip for the isolation and purification of aloperine (ALP), sophoridine (SRI), matrine (MT) and oxymatrine (OMT). The chip has the advantages of high efficiency, reproducibility and applicability for subsequent concentration determination, and is a promising technique for drug quality control.	[62]

**Table 3:** Potentially toxic components and toxic effects of biological pharmaceuticals.

Herb Name	Potentially Toxic Ingredient	Potential Toxic Effects
Ginseng	Ginsenoside Rb₁	Embryotoxicity
Panax ginseng	Ginseng total saponin	Hepatotoxicity
He Shou Wu, cassia seed, senna leaf, rhubarb	Anthraquinone components	Hepatotoxicity, nephrotoxicity, enterotoxicity
Bitter almond, peach kernel, yu li ren	Bitter amygdalin	Cyanide toxicity
Tetradium ruticarpum	Evodiamine, Rutecarpine	Hepatotoxicity
Menthahaplocalyx	Menthol	Brain damage
Tribulus terrestris L.	Tribulus terrestris saponin	Hepatotoxicity

## Data Availability

Not applicable.
